# Delineation of loci governing an extra‐earliness trait in lentil (*Lens culinaris* Medik.) using the QTL‐Seq approach

**DOI:** 10.1111/pbi.14415

**Published:** 2024-06-25

**Authors:** Kumbarahally Murthigowda Shivaprasad, Harsh K. Dikshit, Gyan Prakash Mishra, Subodh Kumar Sinha, Muraleedhar Aski, Manju Kohli, Dwijesh C. Mishra, Amit Kumar Singh, Soma Gupta, Akanksha Singh, Kuldeep Tripathi, Ranjeet Ranjan Kumar, Atul Kumar, Girish Kumar Jha, Shiv Kumar, Rajeev K. Varshney

**Affiliations:** ^1^ Division of Genetics Indian Agricultural Research Institute New Delhi India; ^2^ Indian Council of Forestry Research and Education (ICFRE)‐Institute of Forest Biodiversity Hyderabad India; ^3^ Indian Council of Agricultural Research (ICAR)‐National Institute for Plant Biotechnology New Delhi India; ^4^ Indian Agricultural Statistics Research Institute New Delhi India; ^5^ Division of Genomic Resources, National Bureau of Plant Genetic Resources New Delhi India; ^6^ South Asia and China Program, International Center for Agricultural Research in the Dry Areas, National Agriculture Science Complex New Delhi India; ^7^ Germplasm Evaluation Division, National Bureau of Plant Genetic Resources New Delhi India; ^8^ Division of Biochemistry Indian Agricultural Research Institute New Delhi India; ^9^ Division of Seed Science and Technology Indian Agricultural Research Institute New Delhi India; ^10^ Centre for Crop & Food Innovation, State Agricultural Biotechnology Centre Food Futures Institute, Murdoch University Murdoch WA Australia

**Keywords:** *Elf3a*, InDel markers, qPCR, QTL mapping, RILs, WGRS

## Abstract

Developing early maturing lentil has the potential to minimize yield losses, mainly during terminal drought. Whole‐genome resequencing (WGRS) based QTL‐seq identified the loci governing earliness in lentil. The genetic analysis for maturity duration provided a good fit to 3:1 segregation (F_2_), indicating earliness as a recessive trait. WGRS of Globe Mutant (late parent), late‐flowering, and early‐flowering bulks (from RILs) has generated 1124.57, 1052.24 million raw and clean reads, respectively. The QTL‐Seq identified three QTLs (*LcqDTF3*.*1*, *LcqDTF3*.*2*, and *LcqDTF3*.*3*) on chromosome 3 having 246244 SNPs and 15577 insertions/deletions (InDels) and 13 flowering pathway genes. Of these, 11 exhibited sequence variations between bulks and validation (qPCR) revealed a significant difference in the expression of nine candidate genes (*LcGA20oxG*, *LcFRI*, *LcLFY*, *LcSPL13a*, *Lcu*.*2RBY*.*3g060720*, *Lcu*.*2RBY*.*3g062540*, *Lcu*.*2RBY*.*3g062760*, *LcELF3a*, and *LcEMF1*). Interestingly, the *LcELF3a* gene showed significantly higher expression in late‐flowering genotype and exhibited substantial involvement in promoting lateness. Subsequently, an InDel marker (I‐SP‐383.9; *LcELF3a* gene) developed from *LcqDTF3*.*2* QTL region showed 82.35% PVE (phenotypic variation explained) for earliness. The cloning, sequencing, and comparative analysis of the *LcELF3a* gene from both parents revealed 23 SNPs and InDels. Interestingly, a 52 bp deletion was recorded in the *LcELF3a* gene of L4775, predicted to cause premature termination of protein synthesis after 4 missense amino acids beyond the 351st amino acid due to the frameshift during translation. The identified InDel marker holds significant potential for breeding early maturing lentil varieties.

## Introduction

Lentil (*Lens culinaris* Medik.) is a diploid (2*n* = 14; 4063 Mbp) legume crop that is cultivated in semi‐arid and temperate regions of the World. Globally, it is cultivated on nearly 5.01 Mha area, yielding 6.54 million metric tons (Mt) with a productivity of 1305 kg/ha. India and Canada collectively account for nearly 61.8% of the World's lentil production. In India, it is grown on a 1.35 Mha area, fetching nearly 1.18 Mt production with 871.5 kg/ha productivity (Dikshit *et al*., 2022b; FAOSTAT, 2020). Lentils are rich in proteins, carbohydrates, fibres, vitamins, and micronutrients, and thus help ensure nutritional security of impoverished populations especially in developing countries (Dikshit *et al*., 2022a; Priti Mishra *et al*., 2021). Untill now, the CDC Redberry Genome Assembly v2.0 is the latest version (pre‐release) of the lentil genome (Ramsay *et al*., 2021) that is publicly available for genomic studies.

In South Asian countries like India, Nepal, Pakistan, and Bangladesh; lentil is predominantly cultivated during the rabi season (October to March). Lentil productivity gets severely hindered by the terminal drought stress, especially in the late‐flowering cultivars. Whereas early maturing cultivars exhibit higher yields as these manage to escape the terminal drought stress (Aski *et al*., 2023a,b; Shrestha *et al*., 2005). Therefore, breeding for earliness in lentils is one of the key traits in the existing climate change scenario. Flowering time greatly influences the lentil yield and adaptability to changing environmental conditions. The mapping of quantitative trait loci (QTLs) regulating early‐flowering in lentil has been the subject of interest for the breeders aiming to develop early‐maturing varieties with an improved yield.

To date, several QTLs regulating days to flowering (DTF) have been identified in lentil. For DTF, three QTLs each have been identified by Tahir and Muehlbauer (1994) and Fratini et al. (2007), while Tullu *et al*. (2008) mapped five QTLs in lentil. However, Kahriman *et al*. (2015) and Polanco *et al*. (2019) have identified one major QTL on LG6 (44–60% PVE) and chromosome 6, respectively. Saha *et al*. (2013) identified three QTLs for days to 50% flowering (15.6–24.2% PVE); while Yuan *et al*. (2021) associated a florigen gene (*LcFTa1*) with a major QTL, which is flowering time sensitive to light quality. Rajandran (2016) identified a QTL, DTF6a having 10 335‐bp deletion between *FTa1* and *Fta2* genes, causing up‐regulation of *FTa1* and promoting early flowering. Conversely, DTF6b QTL involves a legume‐specific paralogue of Arabidopsis *PSEUDO‐RESPONSE REGULATOR* (*PRR59c*), contributing to earliness in lentil (Rajandran *et al*., 2022).

The association mapping in lentil revealed 13 linked EST‐SSR markers with flowering time, and the PBALC0250 marker explained 21.8% PVE (Kumar *et al*., 2018). Genome‐wide association analysis (GWAS) using 176 ICARDA (International Center for Agriculture Research in the Dry Areas) lentil reference set Plus collection has identified two Marker‐Trait Associations (MTAs) for DTF and 08 MTAs for days to maturity (DTM) (Rajendran *et al*., 2021).

Sarker *et al*. (1999) reported that the early flowering in lentil is regulated by *Sn* locus which is linked to seed coat colour gene *Scp*. However, in peas, the earliness is reportedly governed by the *HIGH RESPONSE TO PHOTOPERIOD* (*Hr*) locus which is an ortholog of the *Arabidopsis ELF3* gene. Interestingly, the *ELF3* orthologs in lentil also showed its correspondence with the pea *Hr* locus. In the *ELF3* locus (an ortholog of pea *Hr* locus), a translationally silent G‐to‐A substitution was recorded in the last nucleotide of exon 3, causing mis‐splicing and exon 3 skipping, which results in a frame‐shift during the translation of exon 4 (Weller *et al*., 2012). However, *ELF3* (*LcELF3a* or *LcELF3b*) was neither identified nor mapped to any lentil chromosomes.

The next‐generation sequencing (NGS) based ‘Quantitative Trait Loci‐Sequencing’ (QTL‐Seq) is a rapid and efficient genome‐wide approach to map the major loci governing quantitative traits. It compares the SNPs between the extreme bulks with any one parent and the reference genome, thereby identifying the QTLs governing the trait of interest (Takagi *et al*., 2013). Despite the importance of extra‐early flowering genotypes in lentil, our understanding of the genetic loci governing this trait and their validation using genomics resources is minimal. Against this backdrop, this study deployed the novel QTL‐Seq approach to map the loci regulating earliness trait in lentil.

## Materials and methods

### Development of mapping population

**Figure 1:**
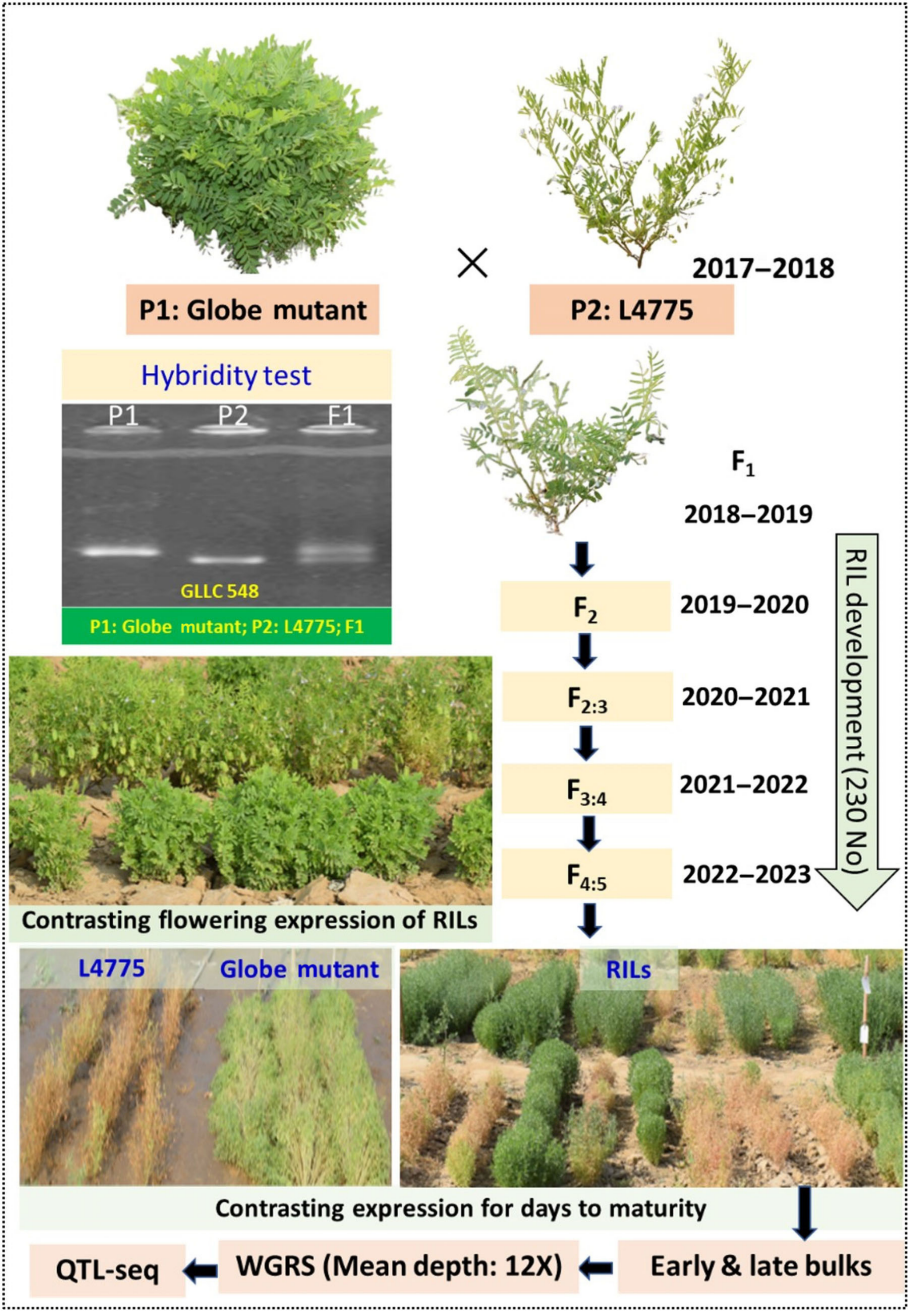
Schematic presentation of RIL development for the QTL‐Seq analysis in lentil.

A large number of lentil genotypes, varieties, and breeding lines were phenotyped for DTF at the research farm of ICAR‐Indian Agriculture Research Institute (IARI, New Delhi, India) located at latitude‐28.08° N, longitude‐77.12° E, and altitude‐228.61 m AMSL during *rabi* 2016–17. The F_1_ was generated by crossing two contrasting genotypes viz., Globe Mutant (90–100 DTF; 135–145 DTM) as female with L4775 (55–65 DTF; 95–105 DTM) as male during 2017–18. The Globe Mutant has a globe‐type plant morphology (recessive), while L4775 has spreading‐type plant morphology (dominant); and as expected, F_1_s exhibited L4775‐type plant morphology (Figure 1). F_1_ was then allowed to self to get the F_2's_ (*rabi* 2018–19) which were again selfed and advanced using a single seed descent (SSD) method to generate F_2:3_ (*rabi* 2019–20), F_3:4_ (*rabi* 2020–21), and F_4:5_ (*rabi* 2021–22) having final 230 RILs.

### Hybridity test

To confirm the hybridity of F_1s_, genomic DNA was isolated using the CTAB method (Murray and Thompson, 1980) from parents (Globe Mutant and L4775) and F_1s_; the hybridity of F_1s_ was confirmed using the polymorphic SSR marker GLLC548 (Saha *et al*., 2010). The polymerase chain reaction (PCR) was performed using a PCR mix of 10 μL consisting of 80 ng of DNA template, 2 × Taq Buffer A, 1 mM dNTP mix, 0.15 μL Taq polymerase (Meridian Bioscience), and 2.5 pmoles each of forward and reverse primers. The cycling conditions for PCR amplification were 94°C (3.0 min), 35 cycles of 94°C (30 s), 55°C (30 s), and 72°C (60 s) for denaturation, annealing, and extension, respectively, followed by a final extension at 72°C (8 min) and the amplified products were separated on a 3.0% agarose gel.

### Phenotyping for flowering time

The plants which matured in more than 110 days were considered late maturing, while those matured in <110 days were considered early maturing. To study the genetics of earliness in lentil, 231 F_2_ plants were grown and phenotyped for DTM at IARI, New Delhi field during *rabi* 2021–2022. The Chi‐square test (Pearson, 1900) was used to test the goodness of fit of the F_2_ ratios of DTM. The observations were also recorded for DTF in F_3:4_, DTF, and DTM in the F_4:5_ populations grown at the IARI field during *rabi* 2021–2022 and *rabi* 2022–2023, respectively. Twenty extreme RILs each having stable expression for very‐early and very‐late maturity in terms of DTF and DTM were selected for QTL‐Seq analysis.

### Extraction of genomic DNA and whole genome resequencing

**Figure 2:**
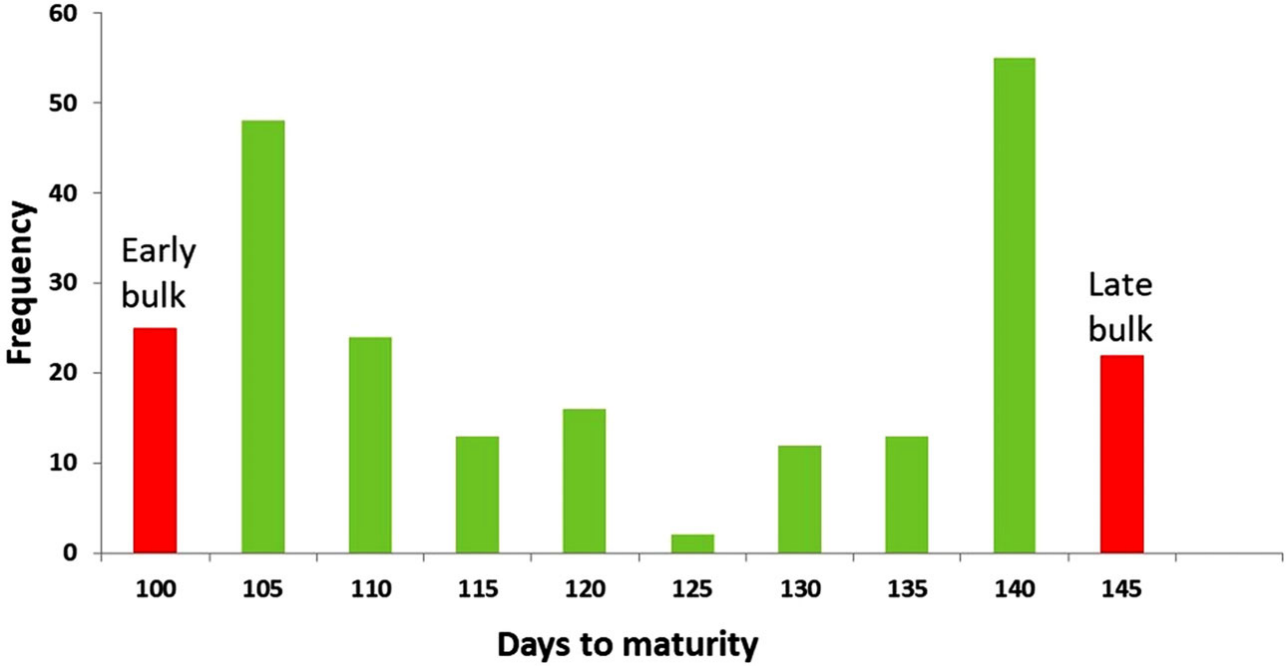
Frequency distribution of mean days to maturity among 230 RILs. The extreme 20 individuals (marked in red) were used to make the early bulk and late bulk for QTL‐Seq analysis.

Genomic DNA was isolated using the CTAB method (Murray and Thompson, 1980) from a parent (Globe Mutant) and 20 extreme RILs (F_4:5_), each having the lowest and highest DTF. An equal quantity of DNA was pooled from the two extreme categories to constitute the early bulk (EB) and late bulk (LB) (Figure 2). The DNA quantity was confirmed using Nanodrop 2000, while quality and integrity were checked on 1% agarose gel. About 1.2 μL of high‐quality genomic DNA per sample was used for library preparation using NEBNext® Ultra™ II DNA Library Prep Kit of Illumina®. Nearly 300–600 bp insert size libraries were processed for paired‐end (PE) sequencing (2 × 151 bp PE) using the Illumina Novaseq 6000 platform (Illumina Technologies, USA), and the raw sequence data were filtered using a standard Illumina pipeline. The sequence is submitted to NCBI sequence read archive (SRA) number PRJNA915231 (https://www.ncbi.nlm.nih.gov/bioproject/PRJNA915231).

### QTL‐Seq analysis

The quality of raw reads was checked using FastQC (Andrews, 2010), adapter and low‐quality reads were trimmed using Trimmomatic (Bolger *et al*., 2014), clean reads were aligned to the CDC Redberry Genome Assembly v2.0 reference genome (Ramsay *et al*., 2021) using BWA‐MEM (Li, 2013), and two bulks and the Globe Mutant BAM files were used to generate BCF file using bcftools mpileup (Li *et al*., 2009). The SNP calling was done using bcftools call (Li *et al*., 2009), and information about SNPs/Indels position was generated (Globe Mutant, LB, and EB) for each base position. The VCF file was used for the QTL‐Seq pipeline (https://github.com/YuSugihara/QTL‐seq; Grüning *et al*., 2018; Sugihara *et al*., 2022; Takagi *et al*., 2013). The SNP index for both the bulks was calculated by comparing them with the Globe Mutant SNPs/Indels (Abe *et al*., 2012; Takagi *et al*., 2013).

The SNP/InDel position having mapping quality <50, and read depth <8 (in both bulks) was filtered, and the ΔSNP index was calculated by subtracting the SNP index of LB from EB. A sliding window size of 4.0 Mb with 100 kb increment was used to evaluate the mean distribution of Δ(SNP‐index) across the chromosomes, and QTLs governing earliness were identified.

### Candidate gene identification and its validation through qRT‐PCR

The curated gene track of JBrowse was used to identify the candidate flowering genes in the identified QTLs (Ramsay *et al*., 2021; Yuan *et al*., 2021; https://knowpulse.usask.ca/jbrowse/Lens‐culinaris/2). The terminal flower buds and fully expanded leaves were collected from the lentil genotypes L4775 (early maturing) and Globe Mutant (very late maturing), and RNA was isolated using the Trizol method (Chomczynski and Sacchi, 1987). The integrity and purity of isolated RNA were determined on 1% agarose gel, and cDNA was synthesized using a Takara primescript 1st strand cDNA synthesis kit. Gene‐specific primers (Table S1) were designed using the NCBI Primer‐BLAST tool (https://www.ncbi.nlm.nih.gov/tools/primer‐blast/), and validation was done by qRT‐PCR (CFX96 Touch Real‐Time System; Bio‐Rad) using the PowerUp SYBR Green Master Mix (Applied Biosystems). The relative gene expression was determined through the ΔΔCT method (Livak and Schmittgen, 2001) using the lentil actin gene (Lcu.2RBY.L011470.1) as an internal control (Sen Gupta *et al*., 2017; Yuan *et al*., 2021). Each reaction was repeated thrice, and data were presented as mean ± SE. The *t*‐test was performed to analyse the significant differences between the samples.

### Validation of identified QTLs using the InDel marker

An InDel marker (I‐SP‐383.9) having a 34‐base deletion in the early‐flowering genotype L4775 was identified from the *LcqDTF3*.*2* QTL region at position 383 964 202 near the *LcELF3a* gene (Table S2). The 400 bp upstream and downstream flanking sequences from the InDel were extracted using the reference genome fasta file and primers were designed using the NCBI Primer‐BLAST tool (https://www.ncbi.nlm.nih.gov/tools/primer‐blast/) (Table S3). PCR was performed using the PCR mix (10 μL) consisting of 80 ng template DNA, 2 × Taq Buffer A, 1.0 mM dNTP mix, 0.75 units of Taq polymerase (5 U/μL, Meridian Bioscience), and 2.5 pmoles each of forward and reverse primers. The cycling conditions were 94°C (3.0 min), 35 cycles of 94°C (30 s), 60°C (30 s), and 72°C (60 s) for extension, followed by final extension at 72°C (10.0 min) and the amplified product was scored using 3.0% agarose gel. The 125 bp and 91 bp bands correspond to late and early parents, respectively. This marker was used for the genotyping of 230 RILs (F_4:5_). Single marker analysis was performed through simple linear regression analyses using genotypic data (considering the InDel marker as an independent variable) and the phenotypic data for DTM of *rabi* 2022–2023 as the dependent variable using MS Excel.

### Cloning of the cDNA *LcELF3a* gene

The cDNA of the *LcELF3a* gene was amplified from both the parents (Globe Mutant and L4775) using newly designed primers (Table S4). The PCR products were eluted using the QIAquick Gel Extraction Kit (QIAGEN, Valencia) and ligated with pJET1.2/blunt vector using the CloneJET PCR Cloning Kit (Thermo Scientific™) (https://www.thermofisher.com/order/catalog/product/K1231). The recombinant vector was then transformed into competent cells (*E*. *coli* DH5α strain) for cloning. Afterward, the plasmid DNA was isolated using the QIAprep Spin Miniprep Kit and stored at −20°C for further analysis. The cloning was confirmed by restriction digestion using XhoI, and positive clones were sequenced through the Sanger sequencing platform using universal primers and the gap primer (Table S4). The raw sequence data were processed by trimming the vector sequence and the sequences were compared using BioEdit (Hall, 1999) and T‐Coffee (Notredame *et al*., 2000).

## Results

### The inheritance of flowering time

The inheritance of DTM was studied in an F_2_ population [Globe Mutant (135–144 DTM) × L4775 (95–105 DTM)]. The 231 F_2_ plants were categorized into two distinct classes: early maturing (<110 DTM; No = 57) and late maturing (>110 DTM; No = 174). Chi‐square analysis (F_2_) demonstrated a single dominant gene controlling flowering time or maturity duration (*P* = 0.91, *χ* ^2^ = 0.013), and late flowering showed dominant, while early flowering showed recessive expression.

### Phenotyping and construction of extreme flowering bulks

A significant difference in the DTM was observed in the studied RILs (F_4:5_) with maturity duration ranging from 96 to 144 days (Mean: 119.82 ± 16.28 d). The DTM showed bimodal distribution in RILs, which indicated earliness under the control of a few major loci (Figure 2). Twenty extreme individuals of the RIL population for flowering time were used to develop EB (DTF mean: 57.8 d, DTF range: 55–60 d; DTM mean: 99.0 d, DTM range: 96–100 d) and LB (DTF mean: 90.6 d, DTF range: 84–98 d, DTM mean: 139.9 d, DTM range: 136–144 d).

### QTL‐Seq analysis using WGRS and mapping of reads

The WGRS data were generated for Globe Mutant (208.12 million reads or 31.42 Gb), LB (462.67 million reads or 69.86 Gb), and EB (453.79 million reads or 68.52 Gb) (Table 1). The raw sequences are submitted to the NCBI as SRA number PRJNA915231 (https://www.ncbi.nlm.nih.gov/sra/PRJNA915231). After filtering the poor‐quality reads and trimming poor‐quality bases, overall, 196.89, 420.81, and 434.53 million clean reads were generated from Globe Mutant (7.3× depth), EB (15.4× depth), and LB (15.9× depth), respectively. About 99.92–99.96% of reads were mapped to unique physical locations of reference lentil genome. The highest number of reads were mapped on chromosome 2, while the lowest were on chromosome 3 (Table 1, Table 2). The mapped reads covered nearly 12.87× mean sequencing depth. After removing the low‐quality SNPs and InDels (including absent and heterozygous sites) in the parental line, 7 444 399 high‐quality SNPs and 385 772 InDels were identified between EB and LB. These SNPs/InDels were further used for identifying the region regulating earliness in lentil using Δ(SNP index) algorithms of the QTL‐Seq pipeline.

**Table 1:** Sequencing and mapping details of a parental line and two bulks for flowering time

Sample	No of lines bulked	Total reads generated (Million)	High‐quality reads (Million)	Reads mapped (Million)	Average depth (×)	Properly paired reads (Million)
Globe Mutant	–	208.12	196.89	196.80 (99.96%)	7.3	184.62 (95.25%)
Late bulk	20	462.67	434.53	428.24 (99.92%)	15.9	415.68 (96.99%)
Early bulk	20	453.78	420.81	420.48 (99.92%)	15.4	399.26 (96.03%)

**Table 2:** Chromosome‐wise details of mapped reads of the parental line and two bulks

Chromosome	Reference sequence length	Number of mapped reads		
		Globe Mutant	Late bulk	Early bulk
Lcu.2RBY.Chr1	538362633	28698189	62819735	60670352
Lcu.2RBY.Chr2	614089212	30378561	67736963	65643294
Lcu.2RBY.Chr3	430115100	20279896	43854753	44290796
Lcu.2RBY.Chr4	482216434	24868656	53989127	52169975
Lcu.2RBY.Chr5	475007465	25184356	54931112	52594182
Lcu.2RBY.Chr6	420584759	21079679	45912900	44344127
Lcu.2RBY.Chr7	529090 306	29521153	67719239	65012833
2068 unplaced unitigs	270231015	16798945	31280569	35755269
Total	3759696924	196809435	428244398	420480828

### Candidate genomic region(s) and candidate genes for extra‐earliness

**Figure 3:**
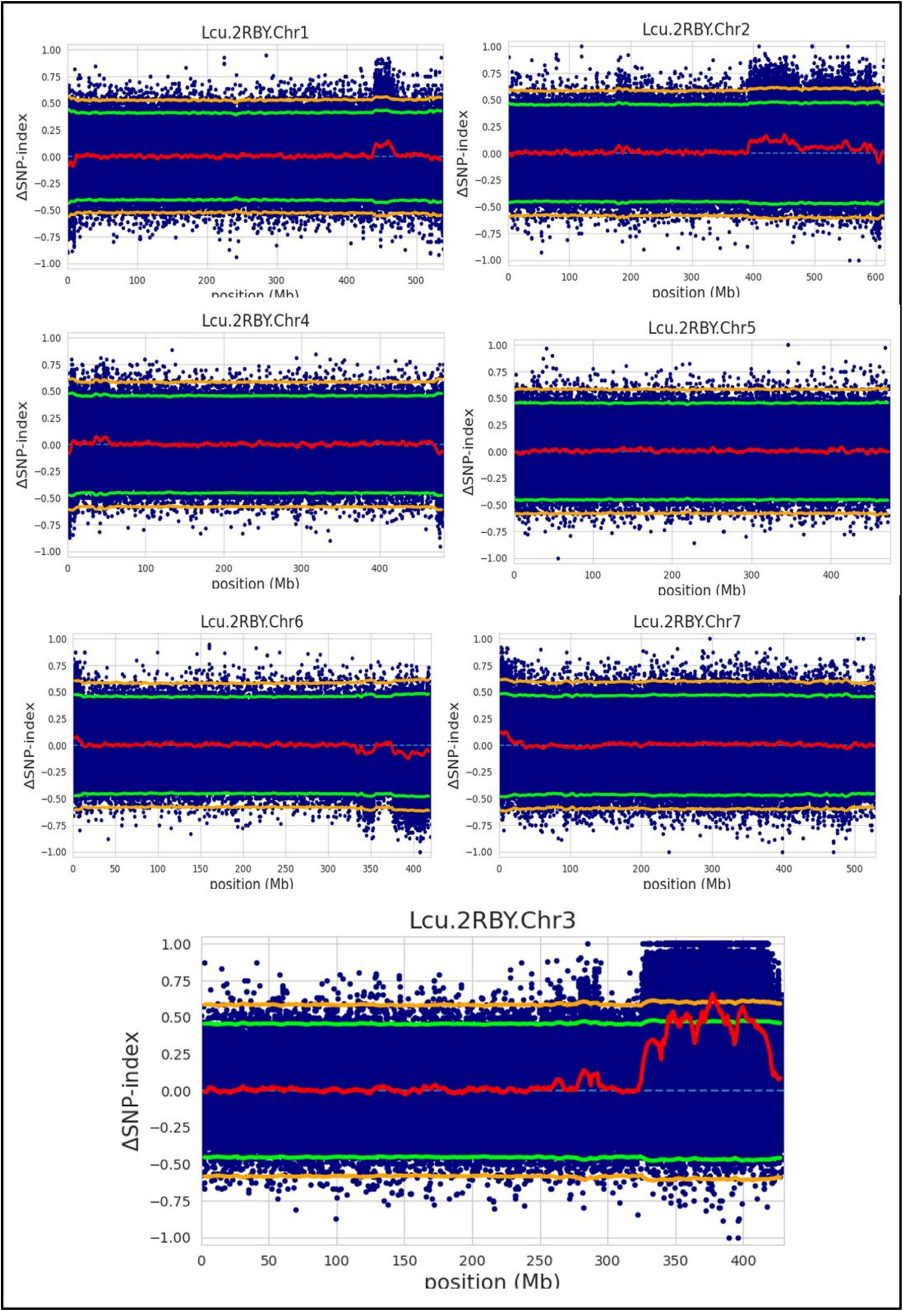
The Δ(SNP‐index) plot illustrates chromosome‐wise details of the ΔSNP index, indicating the presence of QTLs exclusively on chromosome 3 of lentil. The X‐axis specifies the physical locations (Mb) of lentil chromosomes, while Y‐axis designates the ΔSNP‐index measured using a 4.0 Mb sliding window with a 100 kb increment (red line). Statistical confidence intervals for the null hypothesis of no QTLs (*P* ≤ 0.05; green line) (*P* ≤ 0.01; orange line).

**Figure 4:**
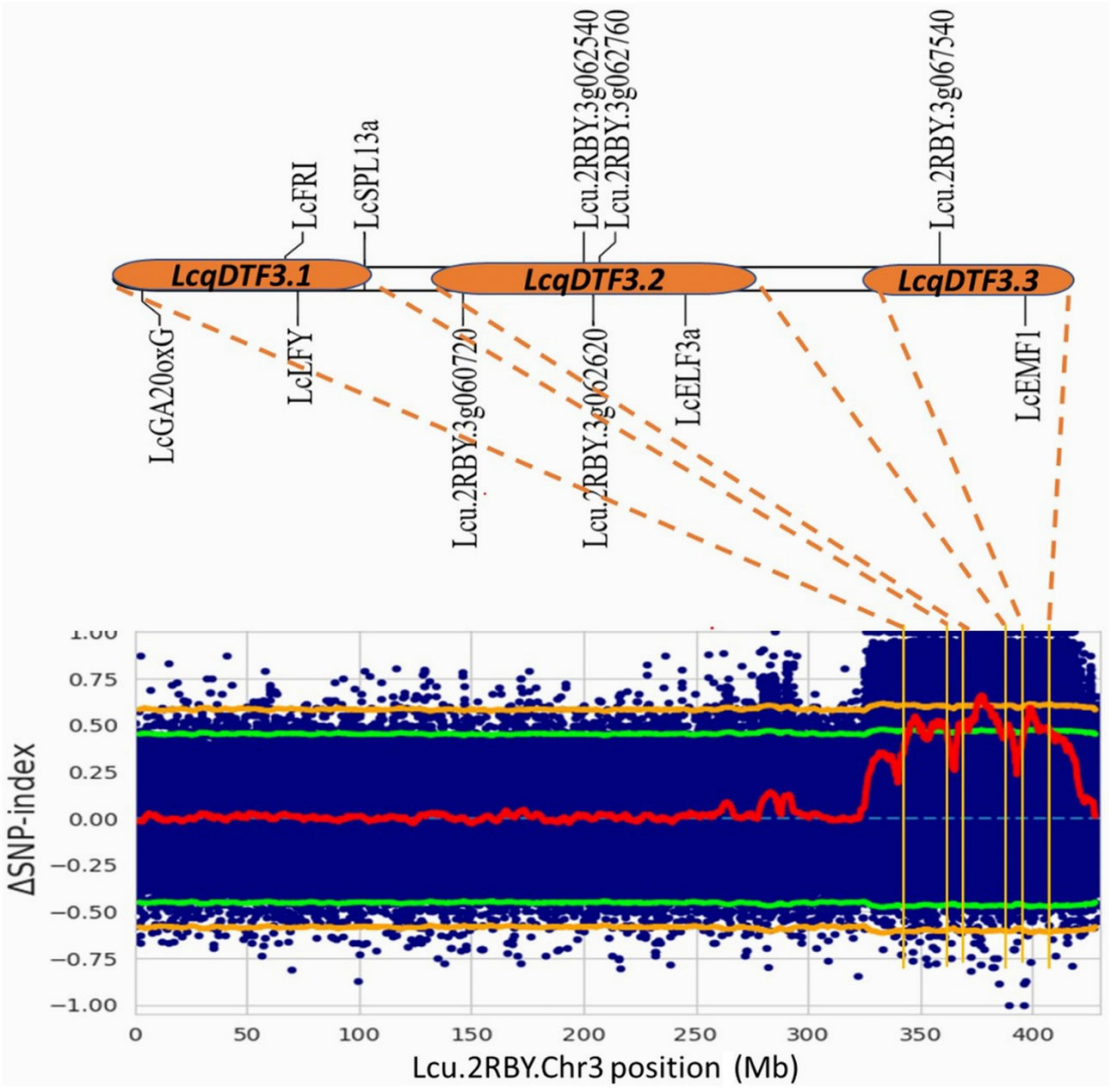
The Δ(SNP‐index) plot of lentil chromosome‐3 showing QTL regions and the candidate genes governing days to flowering (DTF) namely, *LcqDTF3*.*1*, *LcqDTF3*.*2*, and *LcqDTF3*.*3*.

The genomic region(s) controlling earliness was identified by calculating the genome‐wide SNP index for each bulk and Δ(SNP‐index), which was then plotted across the lentil reference genome (Figure 3). For earliness, three major significant genomic regions (*LcqDTF3*.*1*: 345300000 to 361900000 bp, *LcqDTF3*.*2*: 366800000 to 386200000 bp, and *LcqDTF3*.*3*: 396300000 to 407200000 bp) were identified on chromosome‐3 (Figure 4, Table 3). These genomic regions contained 246244 SNPs and 15577 InDels with mapping quality ≥50 and a read depth of ≥8. Based on the reference genome annotation (https://knowpulse.usask.ca/jbrowse/Lens‐culinaris/2; Ramsay *et al*., 2021), 13 flowering pathway genes were identified in these identified QTL regions.

**Table 3:** The position details of three QTLs as identified by the QTL‐Seq approach

QTLs	Start site	End site	Length (Mb)	SNPs	InDels
*LcqDTF3*.*1*	345300000	361900000	16.6	80264	4900
*LcqDTF3*.*2*	366800000	386200000	19.4	99912	6685
*LcqDTF3*.*3*	396300000	407200000	10.9	66068	3992
Total	–	–	46.9	246244	15577

The first QTL (*LcqDTF3*.*1*) has 5 flowering genes [*LcGA20oxG* (*GA20‐oxidase*), *LcFRI* (*Frigida*), *LcLFY* (*LEAFY*), *LcSPL13a* (*SQUAMOSA PROMOTER BINDING PROTEIN‐LIKE*), *and Lcu*.*2RBY*.*3g059660* (nuclear transcription factor Y subunit C3)]; while the second QTL (*LcqDTF3*.*2*) contains 6 flowering genes [*Lcu*.*2RBY*.*3g060720* (*SUPPRESSOR OF PHYTOCHROME A‐105 1*/Ubiquitin ligase cop1), *Lcu*.*2RBY*.*3g062540* (*SLEEPY1*/F‐box GID2‐like protein), *Lcu*.*2RBY*.*3g062620* (*NF‐YA7*/CCAAT‐binding transcription factor), *Lcu*.*2RBY*.*3g062640* (CCAAT‐binding transcription factor), *Lcu*.*2RBY*.*3g062760* (*AGL* MADS‐box transcription factor), and *LcELF3a* (*EARLY FLOWERING 3 like gene*)]. The third QTL (*LcqDTF3*.*3*) harbours only two flowering‐related genes *viz*., *Lcu*.*2RBY*.*3g067540* (Ubiquitin carboxyl‐terminal hydrolase) and *LcEMF1* (*EMBRYONIC FLOWER*) (Table S5).

These candidate genes harboured 134 effective SNPs/InDels in the coding (synonymous and non‐synonymous) and non‐coding intronic regions. Of these, 14 SNPs in *LcGA20oxG*, 18 SNPs and 01 InDel in *LcFRI*, 11 SNPs and 1 InDel in *LcLFY*, 2 SNPs in *LcSPL13a*, 1 SNP in *Lcu*.*2RBY*.*3g060720*, 4 SNPs and 2 InDels in *Lcu*.*2RBY*.*3g062540*, 13 SNPs in *Lcu*.*2RBY*.*3g062620*, 01 InDel and 3 SNPs in *Lcu*.*2RBY*.*3g062760*, 38 SNPs and 5 InDels in *LcELF3a*, 16 SNPs in *Lcu*.*2RBY*.*3g067540*, while 1 InDel and 4 SNPs in *LcEMF1* genes were identified regulating flowering time in lentil (Table 4). The detailed mutation analysis of candidate genes showed stop‐gained variations in the *LcFRI* gene, the splice region variant in the *LcELF3a* gene, and the stop‐lost and splice region variant in *Lcu*.*2RBY*.*3g062620* genes having a significant effect on the proteins encoded along with other missense variants.

**Table 4:** Identification of SNPs in putative candidate genes in the genomic region for early flowering on lentil chromosome‐3 (Lcu.2RBY.Chr3)

QTL	Gene id	Position (bp)	Globe Mutant base	LB base	EB base	ΔSNP index	Reference allele	Alternate allele	Effect type with respect to alternate allele
LcqDTF3.1	*LcGA20oxG (Lcu*.*2RBY*.*3g057140)*	347073988	C	C	T	1	T	C	Intron_variant
		347074011	A	A	C	1	C	A	Intron_variant
		347074031	T	T	C	1	C	T	Intron_variant
		347074078	T	T	C	1	C	T	Intron_variant
		347074094	T	T	C	1	C	T	Intron_variant
		347074100	G	G	A	1	A	G	Intron_variant
		347074284	T	T	C	1	C	T	Intron_variant
		347074818	G	G	A	1	A	G	Synonymous_variant
		347074851	A	A	C	1	C	A	Intron_variant
		347074911	T	T	A	1	A	T	Intron_variant
		347074918	C	C	T	1	T	C	Intron_variant
		347074924	T	T	G	1	G	T	Intron_variant
		347074951	C	C	T	1	T	C	Intron_variant
		347075189	C	C	T	1	T	C	Synonymous_variant
LcqDTF3.1	*LcFRI (Lcu*.*2RBY*.*3g058570)*	356509027	G	G	A	1	A	G	3_prime_UTR_variant
		356509033	T	T	C	1	C	T	3_prime_UTR_variant
		356509132	A	A	C	1	C	A	3_prime_UTR_variant
		356509175	G	G	T	1	T	G	3_prime_UTR_variant
		356509277	A	A	C	1	C	A	3_prime_UTR_variant
		356509537	T	T	C	1	C	T	Missense_variant
		356510200	T	T	A	1	A	T	Intron_variant
		356510293	T	T	G	1	G	T	Stop_gained
		356512108	A	A	T	1	T	A	Synonymous_variant
		356512527	A	A	T	1	T	A	Missense_variant
		356512569	C	C	T	1	T	C	Missense_variant
		356512581	C	C	G	1	G	C	Missense_variant
		356512647	G	G	A	1	A	G	Missense_variant
		356512713	C	C	G	1	G	C	Missense_variant
		356512819	A	A	G	1	G	A	Synonymous_variant
		356512892	A	A	G	1	G	A	Missense_variant
		356512977	G	G	C	1	C	G	5_prime_UTR_variant
		356513068	A	A	G	1	G	A	5_prime_UTR_premature_start_codon_gain_variant
LcqDTF3.1	*LcLFY (Lcu*.*2RBY*.*3g058810)*	357350930	T	T	C	1	C	T	Downstream_gene_variant
		357351319	T	T	C	1	C	T	Missense_variant
		357351403	A	A	G	1	G	A	Missense_variant
		357351406	G	G	A	1	A	G	Missense_variant
		357351427	C	C	T	1	T	C	Missense_variant
		357351463	C	C	T	1	T	C	Missense_variant
		357351592	T	T	C	1	C	T	Missense_variant & splice_region_variant
		357351603	A	A	G	1	G	A	Downstream_gene_variant
		357351643	G	G	C	1	C	G	Downstream_gene_variant
		357351691	C	C	A	1	A	C	Downstream_gene_variant
		357351737	AAT	AAT	AATGTAGAGTCCAT	1	AATGTAGAGTCCAT	AAT	Downstream_gene_variant
		357351784	G	G	A	1	A	G	Downstream_gene_variant
LcqDTF3.1	*LcSPL13a (Lcu*.*2RBY*.*3g059590)*	361573172	A	A	G	1	G	A	Missense_variant
		361574000	G	G	A	1	A	G	Intron_variant
		361575042	TAAAAAAAAA	TAAAAAAAAA	TAAAAAAAAAA	1	TAAAAAAAAAA	TAAAAAAAAA	Intron_variant
LcqDTF3.2	*Lcu*.*2RBY*.*3g060720*	368144638	A	A	G	1	G	A	Missense_variant
LcqDTF3.2	*Lcu*.*2RBY*.*3g062540*	375998977	T	T	C	1	C	T	3_prime_UTR_variant
		375998989	TGG	TGG	TG	1	TG	TGG	3_prime_UTR_variant
		375999347	CATAATAATA	CATAATAATA	CATAATA	1	CATAATA	CATAATAATA	3_prime_UTR_variant
		375999529	A	A	G	1	G	A	3_prime_UTR_variant
		375999705	A	A	T	1	T	A	3_prime_UTR_variant
		376000249	G	G	A	1	A	G	Synonymous_variant
LcqDTF3.2	*Lcu*.*2RBY*.*3g062620*	376524158	T	T	C	1	C	T	Stop_gained
		376524179	C	C	T	1	T	C	Missense_variant
		376524212	A	A	G	1	G	A	Missense_variant
		376524243	G	G	A	1	A	G	Stop_lost
		376524244	C	C	T	1	T	C	Missense_variant
		376524263	C	C	T	1	T	C	Splice_region_variant&intron_variant
		376524319	G	G	A	1	A	G	Missense_variant
		376524330	C	C	T	1	T	C	Missense_variant
		376524332	G	G	A	1	A	G	Synonymous_variant
		376524351	A	A	G	1	G	A	Missense_variant
		376524672	T	T	C	1	C	T	Synonymous_variant
		376524705	C	C	T	1	T	C	Stop_lost&splice_region_variant
		376524706	A	A	G	1	G	A	Splice_region_variant&stop_retained_variant
LcqDTF3.2	*Lcu*.*2RBY*.*3g062760*	376970468	A	A	G	1	G	A	Synonymous_variant
		376970826	C	C	T	1	T	C	Synonymous_variant
		376972111	C	C	G	1	G	C	Missense_variant
LcqDTF3.2	*LcELF3a (Lcu*.*2RBY*.*3g063730)*	382530511	T	T	C	1	C	T	5_prime_UTR_variant
		382530591	A	A	G	1	G	A	5_prime_UTR_premature_start_codon_gain_variant
		382530662	TA	TA	TAA	1	TAA	TA	5_prime_UTR_variant
		382530932	C	C	A	1	A	C	Downstream_gene_variant
		382531063	C	C	T	1	T	C	Downstream_gene_variant
		382531069	G	G	A	1	A	G	Downstream_gene_variant
		382531129	TTTGT	TTTGT	TT	1	TT	TTTGT	Downstream_gene_variant
		382531169	A	A	G	1	G	A	Splice_region_variant&synonymous_variant
		382531199	G	G	T	1	T	G	Synonymous_variant
		382531316	C	C	T	1	T	C	Synonymous_variant
		382531337	C	C	T	1	T	C	Synonymous_variant
		382531562	T	T	C	1	T	C	Synonymous_variant
		382531566	T	T	G	1	G	T	Missense_variant
		382531709	T	T	A	1	A	T	Missense_variant
		382531786	T	T	C	1	C	T	Missense_variant
		382531837	G	G	A	1	A	G	Missense_variant
		382531865	T	T	C	1	C	T	Synonymous_variant
		382531898	TGAC	TGAC	T	1	TGAC	T	Conservative_inframe_deletion
		382532033	A	A	T	1	T	A	Downstream_gene_variant
		382532064	C	C	T	1	T	C	Downstream_gene_variant
		382532166	C	C	T	1	C	T	Downstream_gene_variant
		382532343	T	T	A	1	T	A	Downstream_gene_variant
		382532424	T	T	G	1	G	T	Downstream_gene_variant
		382532500	T	T	A	1	T	A	Downstream_gene_variant
		382532626	T	T	G	1	G	T	Downstream_gene_variant
		382532821	T	T	A	1	T	A	Downstream_gene_variant
		382532857	T	T	G	1	G	T	Downstream_gene_variant
		382532940	TA	TA	T	1	T	TA	Downstream_gene_variant
		382532964	T	T	A	1	T	A	Downstream_gene_variant
		382533015	T	T	C	1	T	C	Downstream_gene_variant
		382533057	G	G	A	1	A	G	Downstream_gene_variant
		382533092	G	G	T	1	T	G	Downstream_gene_variant
		382533104	G	G	A	1	A	G	Downstream_gene_variant
		382533108	TCCG	TCCG	T	1	T	TCCG	Downstream_gene_variant
		382533281	G	G	A	1	G	A	Missense_variant&splice_region_variant
		382533326	A	A	G	1	A	G	Downstream_gene_variant
		382533340	T	T	C	1	C	T	Downstream_gene_variant
		382533351	C	C	G	1	G	C	Downstream_gene_variant
		382534010	A	A	G	1	G	A	Missense_variant
		382534096	G	G	T	1	G	T	Missense_variant
		382534115	C	C	T	1	C	T	Missense_variant
		382534143	T	T	C	1	C	T	Synonymous_variant
		382534377	C	C	T	1	T	C	Synonymous_variant
LcqDTF3.3	*Lcu*.*2RBY*.*3g067540*	399107440	C	C	T	1	T	C	3_prime_UTR_variant
		399107445	A	A	G	1	G	A	3_prime_UTR_variant
		399107493	A	A	T	1	T	A	3_prime_UTR_variant
		399107728	G	G	C	1	C	G	3_prime_UTR_variant
		399107783	A	A	G	1	G	A	3_prime_UTR_variant
		399107826	A	A	T	1	T	A	3_prime_UTR_variant
		399108043	T	T	C	1	C	T	3_prime_UTR_variant
		399108044	G	G	A	1	A	G	3_prime_UTR_variant
		399108110	A	A	G	1	G	A	3_prime_UTR_variant
		399108116	C	C	T	1	T	C	3_prime_UTR_variant
		399108122	A	A	T	1	T	A	3_prime_UTR_variant
		399108128	T	T	C	1	C	T	3_prime_UTR_variant
		399108130	T	T	C	1	C	T	3_prime_UTR_variant
		399108131	C	C	T	1	T	C	3_prime_UTR_variant
		399108133	A	A	G	1	G	A	3_prime_UTR_variant
		399108139	T	T	C	1	C	T	3_prime_UTR_variant
LcqDTF3.3	*LcEMF1 (Lcu*.*2RBY*.*3g068830)*	404789751	C	C	T	1	T	C	Intron_variant
		404791743	G	G	C	1	C	G	3_prime_UTR_variant
		404792485	T	T	C	1	C	T	3_prime_UTR_variant
		404794376	T	T	G	1	G	T	3_prime_UTR_variant
		404794569	ATT	ATT	AT	1	AT	ATT	3_prime_UTR_variant

### Validation of candidate genes through qRT‐PCR and InDel marker analysis

**Figure 5:**
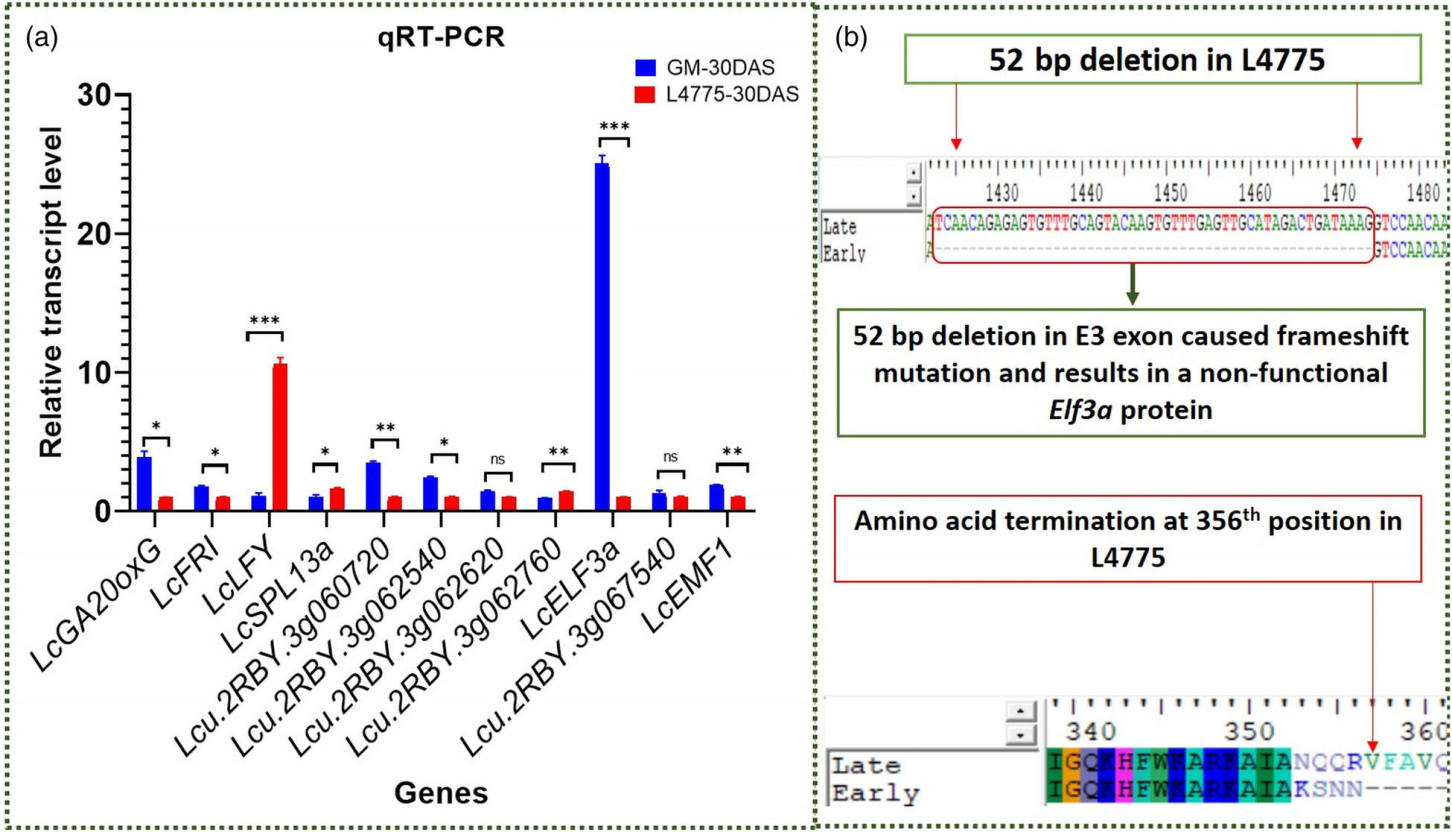
(a) Relative expression of 11 genes in the parental genotype Globe Mutant and L4775. (b) Sequence details of the premature termination in early (L4775) *LcElf3a* protein after 04 missense amino acids beyond the 351st amino acid due to the 52 bp deletion in its transcript. ***P ≤ 0.001, **P ≤ 0.01 and *P ≤ 0.05, based on the *t*‐test.

The expression levels of 11 candidate genes were studied using qRT‐PCR in the parental genotypes Globe Mutant and L4775 (Figure 5a; Table S6). The expression of 6 genes *viz*., *LcFRI* (*P* = 0.005856), *LcEMF1* (*P* = 0.000292), *LcELF3a* (*P* = 0.000002), *Lcu*.*2RBY*.*3g062540* (*P* = 0.000308), *LcGA20oxG* (*P* = 0.003392), and *Lcu*.*2RBY*.*3g060720* (*P* = 0.000043) showed significant upregulation in Globe Mutant over L4775. However, 03 genes, namely, *LcSPL13a* (*P* = 0.030444), *Lcu*.*2RBY*.*3g062760* (*P* = 0.001737), and *LcLFY* (*P* = 0.000058) showed significant upregulation in L4775 than in the Globe Mutant. Interestingly, no significant difference was recorded for *Lcu*.*2RBY*.*3g062620* (*P* = 0.071738) and *Lcu*.*2RBY*.*3g067540* (*P* = 0.240363).

An InDel marker (I‐SP‐383.9), located near the *LcELF3a* gene (1.43 Mb) in a QTL (*LcqDTF3*.*2*) at position 383 964 202 on chromosome‐3 amplified the 125 bp band in the Globe Mutant and the 91 bp band in L4775 genotypes (34 bp InDel). When tested in the RIL, this marker showed perfect co‐segregation with the flowering trait (Figure S1). Single marker analysis using simple linear regression with two classes (EE/Ee = 1 and ee = 0) showed 82.35% PVE for this InDel marker. Conversely, in a three‐class scenario (EE = 1, Ee = 2, and ee = 0), the marker still accounted for 60.57% PVE (Table S7). Other InDels that are identified either in or near the *LcELF3a* gene are of very small size (<20 bp), so these cannot be used to develop a PCR base marker.

### Cloning and sequencing of cDNA of *LcELF3a* gene

The *LcELF3a* cDNA from both parents when cloned in pJET1.2 vector and sequenced revealed 23 SNPs/InDels (Table S8) and 52 bp deletion in early genotype L4775 (2501 bp), which is due to the loss of third exon during the splicing process. This is likely to induce a frameshift during the translation of exon‐4, which may cause premature termination of translation after 04 missense amino acids beyond the 351st amino acid in the protein. In addition, L4775 exhibited 04 missense amino acids, and aspartic acid at position 314 was found deleted. In contrast, the cloned cDNA of *LcELF3a* gene in the late genotype Globe Mutant is 2548 bp long and showed one 6‐bp and two 1‐bp deletions (Figure 5b; Tables S8, S9).

## Discussion

Under the changing climatic conditions, developing early maturing cultivars need chromosome‐based linked genetic markers for earliness. Recent advancements in sequencing have made it possible to use QTL‐seq to identify genomic regions governing earliness traits in lentil (Dutta *et al*., 2022, 2023).

### Flowering time inheritance, mapping population phenotyping, and extreme bulk construction

The F_2_ analysis [Globe Mutant (≥135 days maturity) × L4775 (≤105 days maturity)] for DTM revealed monogenic (recessive) nature of earliness trait. Similar results are reported in other pulses like lentil (Sarker *et al*., 1999), chickpeas (Kumar and Van Rheenen, 2000; Sundaram *et al*., 2019), and peas (Murfet, 1971). However, broad phenotypic variation with bimodality for DTM among studied RILs (F_4:5_) suggested the presence of a few major loci controlling this trait. Similar bimodal expression has been reported in a lentil F_2_ (ILL4605 × ILL6037) population for earliness (Sarker *et al*., 1999). Interestingly, the subsequent RIL (F_4:5_) analysis using SNPs revealed the presence of three tightly linked QTLs on chromosome 3 regulating earliness. This is the power of the QTL‐Seq approach and the SNP markers which could identify three loci on the same chromosome governing earliness in lentil. Similarly, a single dominant gene and a few QTLs governing salinity‐stress tolerance in lentil were reported in a F_2_ population (Singh *et al*., 2020).

The flowering time in legumes is governed by a diverse array of genes (Andrés and Coupland, 2012; Song *et al*., 2013; Weller and Ortega, 2015) including day‐length insensitivity as reported in lentil (Erskine *et al*., 1994) and peas (Arumingtyas and Murfet, 1994). To uncover the genetic loci controlling earliness in lentil, QTL‐seq was conducted using RIL‐derived bulks (20 plants each) with 40–44 days of difference in DTM. Similarly, in cucumber, the F_2_ bulks (10 plants each) with 7–9 days of difference in DTM (Lu *et al*., 2014), in chickpeas, the RIL derived bulks (10 plants each) with 45–64 days of difference in DTM (Srivastava *et al*., 2017), while in pigeon pea, F_2_ bulks (15 plants each) with 27–36 days difference in DTM (Singh *et al*., 2022) were used for QTL‐Seq analysis.

### QTL‐Seq analysis using WGRS and mapping of reads

The WGRS of lentil samples has generated 462.67 and 453.79 million reads for LB and EB, which is 15.9 and 15.4 times the total lentil genome, respectively. A similar level of sequencing depth was also reported for cucumber (Lu *et al*., 2014), broccoli × cabbage (Shu *et al*., 2018), and pigeonpea (Singh *et al*., 2022) for QTL‐seq analysis. Of total reads, nearly 99.92–99.96% were mapped to unique physical locations on the lentil reference genome. Similarly, 86.5–90.1% of reads could be mapped on the reference genome in chickpeas (Srivastava *et al*., 2017). A total of 7 444 399 high‐quality SNPs and 385 772 InDels were identified, and using the ΔSNP index (sliding window‐size: 4 Mb; increment: 100 kb) QTLs regulating earliness are identified on lentil chromosome‐3. Similarly, QTL‐seq has identified 234 393 SNPs in cucumber (sliding window‐size: 1 Mb; increment: 10 kb; Lu *et al*., 2014) and 1 635 117 SNPs in chickpeas (sliding window‐size: 10 Mb; increment: 1 kb; Srivastava *et al*., 2017) for the identification of QTLs regulating earliness.

### QTLs and candidate genes regulating earliness in lentil

The QTL‐seq has identified 3 novel QTLs on lentil chromosome‐3, namely, *LcqDTF3*.*1* (345.3–361.9 Mb), *LcqDTF3*.*2* (366.8–382.6 Mb), and *LcqDTF3*.*3* (396.3–407.2 Mb). Similarly, the GWAS in lentil using the ICARDA reference plus collection has identified 2 MTAs for DTF and DTM on chromosome 3 (Rajendran *et al*., 2021). Also, in cucumber and broccoli × cabbage cross, early flowering QTLs *Ef1*.*1* (22.86–26.31 Mb) and *Ef2*.*1* (0.9–2.9 Mb) were identified on chromosome‐1 (Lu *et al*., 2014) and chromosome‐2 (Shu *et al*., 2018), respectively. Similarly, in chickpeas, two QTLs *CaqbDTF4*.*1* (26.50–27.40 Mb) and *CaqaDTF4*.*2* (46.02–46.78 Mb) were identified controlling DTF on chromosome‐4 (Srivastava *et al*., 2017).

**Figure 6:**
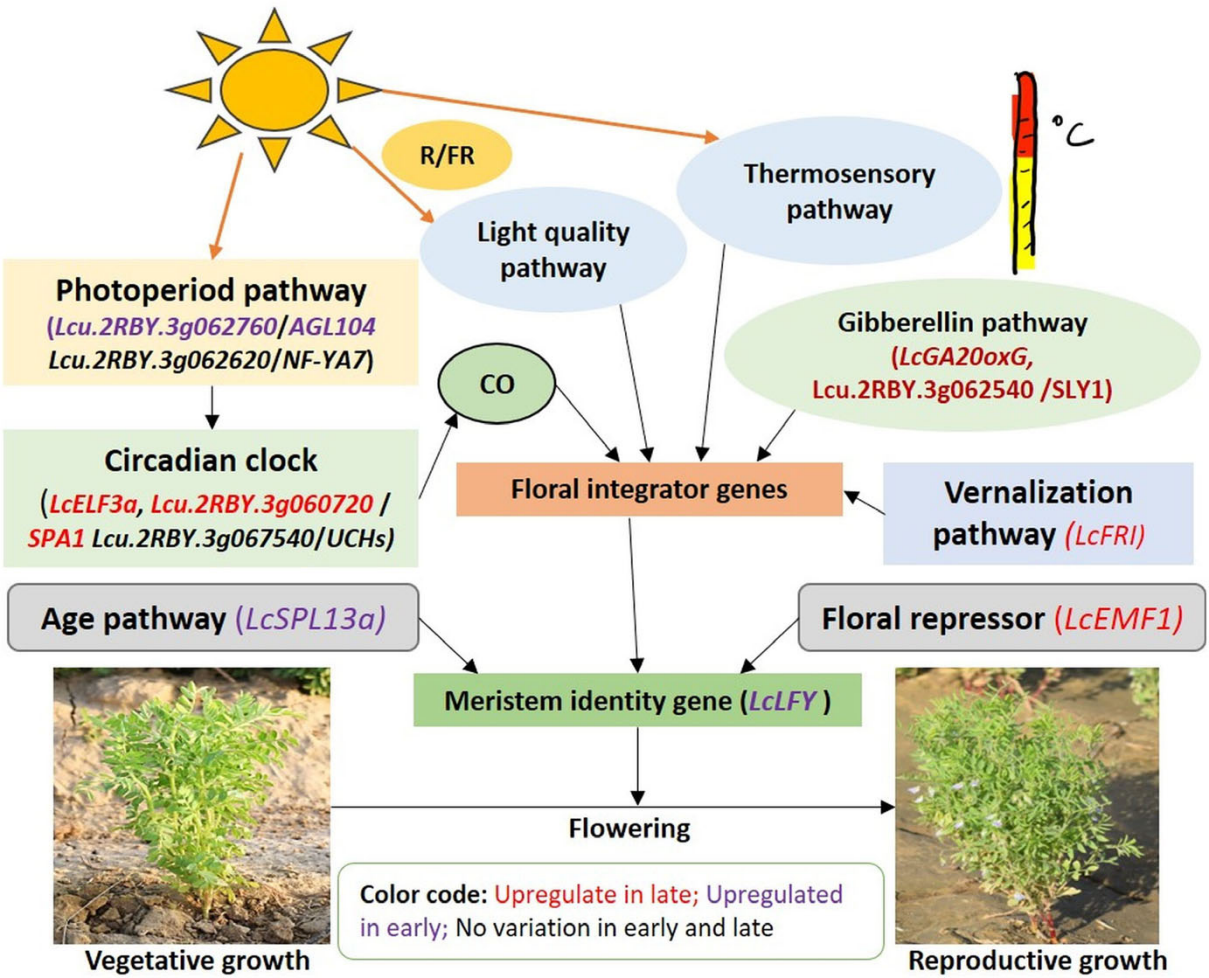
A detailed flowering pathway in lentil shows some key regulators on chromosome 3, governing early flowering in lentil. The picture is derived based on QTL‐Seq and expression analysis results. The late flowering genotype (Globe Mutant) showed upregulation of some flowering‐delaying genes like floral repressor gene, vernalization pathway gene, photoperiod‐circadian clock pathway genes, and gibberellin pathway genes; while the flowering‐promoting genes of the age pathway, photoperiod‐circadian clock, and meristem identity pathway were found downregulated. Conversely, in early‐flowering genotype (L4775), a loss of function of *LcElf3a* (photoperiod‐circadian clock pathway gene) gene was observed regulating early flowering in lentil.

The first QTL (*LcqDTF3*.*1*) showed novel allelic variants in 4 candidate genes (*LcGA20oxG*, *LcFRI*, *LcLFY*, and *LcSPL13a*); and second QTL (*LcqDTF3*.*2*) harboured 5 candidate genes (*Lcu*.*2RBY*.*3g060720*, *Lcu*.*2RBY*.*3g062540*, *Lcu*.*2RBY*.*3g062620*, *Lcu*.*2RBY*.*3g062760*, *LcELF3a*), while third QTL (*LcqDTF3*.*3*) harboured 2 candidate genes (*Lcu*.*2RBY*.*3g067540*, *LcEMF1*) having a Δ(SNP‐index) of 1. Based on QTL‐seq and qRT‐PCR analysis, a detailed flowering pathway is proposed for lentil involving 11 candidate genes (Figure 6). In the late‐flowering genotype (Globe Mutant), conspicuous upregulation of various flowering‐delaying genes like floral repressor gene (*LcEMF1*), the vernalization pathway gene (*LcFRI*), photoperiod‐circadian clock pathway genes (*LcElf3a*, *Lcu*.*2RBY*.*3g060720*), and gibberellin pathway genes (*LcGA20oxG* and *Lcu*.*2RBY*.*3g062540*) were observed. Simultaneously, the downregulation of flowering‐promoting genes of various pathways like the age pathway (*LcSPL13a)*, the photoperiod‐circadian clock pathway (*Lcu*.*2RBY*.*3g062760*), and the meristem identity gene (*LcLFY)* were also recorded in the late flowering lentil genotype (Globe Mutant).

Interestingly, various candidate genes have been identified‐like *LcGA20oxG*, *LcFRI*, *LcLFY*, *LcSPL13a*, *SPA1*, *NF‐YA7*, *AGL104*, *UBP*, *LcEMF*, and *LcELF3* (all on chromosome 3); influencing flowering time in various plant species is thoroughly discussed in this section (Andrés *et al*., 2014; Plackett *et al*., 2012; Tenreira *et al*., 2017; Yuan *et al*., 2021). The up‐regulation of *LcGA20oxG* gene in lentil in response to the low red/far‐red ratio causes stimulation of flowering (Yuan et al., 2021), while gibberellins suppress the flowering in apples (Zhang *et al*., 2019). Significantly higher *LcGA20oxG* gene expression (qRT‐PCR) was recorded in the late flowering genotype Globe Mutant. Similarly, the overexpressing *GA20ox* gene resulted in late flowering in transgenic tomatoes (García‐Hurtado *et al*., 2012) and in grapevines when treated with GA (Boss and Thomas, 2002). Within the *LcGA20oxG* gene, 14 SNP variants (12 intron variants +2 synonymous variants) have been identified as playing a probable role in the regulation of flowering time in lentil.

The *FRI* gene elevates the expression of the *FLOWERING LOCUS C* (*FLC*) gene (Henderson *et al*., 2003) and thereby regulates flowering time in *Brassica rapa* L. (Takada *et al*., 2019) and *Arabidopsis* (Geraldo *et al*., 2009; Le Corre *et al*., 2002). The qRT‐PCR showed significantly higher expression of *LcFRI* gene in the late flowering genotype Globe Mutant. Interestingly, the *LcFRI* gene harboured 18 SNPs, of which an SNP (T) at position 356 510 293 in the Globe Mutant, instead of SNP (G) in L4775, resulted in the acquisition of a stop codon, and thereby formation of a non‐functional *LcFRI* protein. Thus, the non‐functional *LcFRI* protein seems to hinder the regulator of flowering time in lentil even with elevated *LcFRI* gene expression. Additional studies are needed to understand the exact function of *LcFRI* protein in the imposition of early flowering in L4775.

The *LFY* gene encodes a transcription factor regulating the transition from vegetative to the reproductive phase (Weigel *et al*., 1992) by activating the expression of *AP1* and *AP2* genes (in shoot apical meristem), and any mutation in these causes delayed flowering (Ding *et al*., 2018; Jin *et al*., 2021; Liu *et al*., 2017; Weigel *et al*., 1992; William *et al*., 2004). Significantly high *LcLFY* gene expression was recorded in the early‐flowering genotype L4775. Similar high‐expression of the *LFY* gene causing early‐flowering is also reported in *Arabidopsis* (Weigel and Nilsson, 1995), rice (He *et al*., 2000), and strawberry (Liu *et al*., 2017). In the *LcLFY* gene, 12 SNPs/InDel‐based allelic variants were recorded, which may be the reason for lower expression of *LcLFY* gene and delayed flowering in the Globe Mutant.

The *SPL* gene (transcription factor) activates the expression of floral meristem identity gene *LFY* (Shikata *et al*., 2009; Wu and Poethig, 2006), and mutation in this gene causes delayed flowering (Aung *et al*., 2015; Cardon *et al*., 1999; Wu and Poethig, 2006). The *LcSPL13a* gene in Globe Mutant had one InDel and two SNPs, which might have resulted in its lower expression and delayed flowering phenotype. Similarly, in Arabidopsis, *SPL13* regulates the switch from cotyledon to the vegetative stage (Martin *et al*., 2010), while its silencing and down‐regulation causes delayed flowering in alfalfa (Gao *et al*., 2018) and tomatoes (Cui *et al*., 2020), respectively.

The *SPA1* (*SUPPRESSOR OF PHYTOCHROME A‐105 1*) gene controls photoperiodic flowering (Ishikawa *et al*., 2006) and mutation in this gene may cause early flowering. The early flowering genotype L4775 has a missense SNP and exhibited downregulation of lentil *SPA1* gene (*Lcu*.*2RBY*.*3g060720*), implying its role in earliness. Similarly, a mutant *SPA1* gene is reportedly causing early flowering in *Arabidopsis* (Laubinger *et al*., 2006). The *SLEEPY1* (*SLY1*) F‐box gene is a positive regulator of gibberellin (GA) signalling, and mutation in this causes delayed flowering (Ariizumi *et al*., 2011). The *Lcu*.*2RBY*.*3g062540* (lentil *SLY1*) gene had five 3′‐UTR variants, one synonymous_variant, and showed higher expression in the Globe Mutant, a late flowering lentil genotype.

*NF‐YA7* is a transcription factor with a conserved DNA‐binding domain that binds to the promoter at the CCAAT sequence, thereby controlling photoperiod‐regulated flowering time (Kumimoto *et al*., 2008; Siefers *et al*., 2009). The identified lentil *NF‐YA7* (*Lcu*.*2RBY*.*3g062620*) gene contains 13 SNPs, of which 3 were of stop gain/lost type. Among these, SNP ‘T’ at position 376 524 158 in the Globe Mutant (‘C’ in L4775) causes a stop codon gain function and thus non‐functional *NF‐YA7* protein formation. However, expression analysis showed no significant difference between the early and late genotypes. Thus, the formation of non‐functional *NF‐YA7* protein seems causing lateness in Globe Mutant. Similarly, in *Arabidopsis*, the NF‐Y family gene promotes flowering only under inductive photoperiodic conditions (Ben‐Naim *et al*., 2006; Kumimoto *et al*., 2008).

Agamous‐like MADS‐box protein (*AGL*) is a transcription factor that regulates flowering time (Reddy, 2007; Wang *et al*., 2021). The lentil *AGL MADS‐box transcription factor* (*Lcu*.*2RBY*.*3g062760*) showed 3 SNPs (1 missense and 2‐synonymous variants) and displayed higher expression in early genotype L4775. The increased expression of *AGL* gene in L4775 may be causing earliness by exerting a downregulating influence on the functional *LcFRI* gene. Similarly, in *Arabidopsis*, the overexpression of *AGL20* gene suppresses the delayed flowering (Lee *et al*., 2000).

The *ubiquitin C‐terminal hydrolase* (*UCH*) and *ubiquitin‐specific processing protease* (*UBP*) protein family genes regulate the flowering time through protein deubiquitination (Hayama *et al*., 2019; Wang *et al*., 2018). In *UBP* mutants, the *CO* transcript was formed very early (over WT), causing increased *FLT* expression and early flowering (Yoshida *et al*., 2009). The expression analysis of the *UCH* gene (*Lcu*.*2RBY*.*3g067540* with 16 SNPs in the 3′ UTR) did not show any significant difference between early and late lentil genotypes; thus its involvement in the regulation of earliness is ruled out.

The *EMBRYONIC FLOWER* (*EMF*) gene (*EMF1* and *EMF2*) maintains vegetative development by repressing flower development (Moon *et al*., 2003; Sung *et al*., 1992). A lentil flowering time gene *LcEMF1* (with 04 SNPs in 3′ UTR and 01 intron variant) showed significantly low expression and thereby early flowering in L4775. Similarly, in *Arabidopsis*, the suppression of *EMF1* reportedly causes early flowering (Aubert *et al*., 2001; Kim *et al*., 2012; Sánchez *et al*., 2009).

### Loss of function of *ELF3* gene causing earliness in lentil

The *ELF3* gene encodes a nuclear protein that interacts with circadian clock genes like *ELF4* and *LUX ARRHYTHMO* (*LUX*), thereby forming a core complex and ensuring the precision and stability of the circadian clock (Nusinow *et al*., 2011). *ELF3* delays the flowering by inhibiting the flowering‐promoting floral integrator gene (*FT*) expression, especially under long nights or short‐day conditions (Fukazawa *et al*., 2021; Saito *et al*., 2012). A flowering time gene *LcELF3a* has been identified as having 43 SNP/InDels and exhibited significant upregulation in the late‐genotype Globe Mutant.

Intriguingly, an SNP having ‘A’ at position 382 533 281 in L4775 resulted in a splice region variant, in contrast to ‘G’ in the Globe Mutant. The cDNA sequencing of *LcELF3a* gene revealed two deletions in L4775 (52 bp and 3 bp) and one 6 bp and two 1 bp deletions in the Globe Mutant. The 52 bp deletion caused premature translational termination (loss of function) in the early genotype (L4775) after 4 missense amino acids beyond 351st amino acid in the *LcELF3a* protein.

The loss‐of‐function mutations in the *ELF3* gene cause early flowering in various species including, *Arabidopsis* (Hicks *et al*., 2001; Yoshida *et al*., 2009), chickpeas (Basu *et al*., 2022; Ridge *et al*., 2017), and soybean (Fang *et al*., 2021). *ELF3* was validated as the underlying gene of early flowering phenotype in *Triticum monococcum* (Alvarez *et al*., 2016), chickpea (Basu *et al*., 2022; Ridge *et al*., 2017), and *Arabidopsis* (Strasser *et al*., 2009). Interestingly in lentils, a similar result was reported for the unmapped *ELF3* gene using the orthologous pea gene (Weller *et al*., 2012). Our result confirmed that the *LcELF3a* gene mapped in 2nd QTL (*LcqDTF3*.*2*) on chromosome‐3 (position; 382 530 247–382 534 690) is the key gene regulating the earliness in lentil.

Furthermore, an InDel marker (I‐SP‐383.9) near the *LcELF3a* gene also showed very strong co‐segregation with the earliness trait (82.35% PVE). This marker holds great promise for its utilization in the marker‐assisted selection (MAS) in the lentil breeding program. Thus, among various candidate genes identified, the non‐functional *LcELF3a* gene seems the major contributor to imparting earliness in L4775. In addition, it is also interesting to note that all the identified candidate genes regulating flowering time in lentil are clustered together in three major QTLs on chromosome 3.

## Conclusions

This study identified the regions governing flowering time in lentil and expanded our understanding of the molecular mechanisms underlying early flowering in lentil. The results confirmed the use of QTL‐seq in lentil for rapid mapping of major QTLs governing flowering time. Besides, it also effectively identified the non‐synonymous/synonymous SNPs and InDels for 11 candidate genes in three major QTLs for DTF. In future, the findings will enable the development of genomics‐assisted early‐maturing lentil genotypes and varieties.

## Funding

This work was supported and funded by the Indian Council of Agricultural Research (ICAR) and the International Center for Agricultural Research in the Dry Areas (ICARDA).

## Conflict of interest

The authors declare that this research was conducted in the absence of any commercial or financial relationships that could be construed as a potential conflict of interest.

## Author contributions

GM, HD, RV, and SK: Conceptualization; KS, SS, MA, MK, KT, AK, and RK: methodology; KS, DM, SG, AKS, and AS: formal analysis; GM, SK, and HD: resources; KS, HD, GJ, and GM: data curation; KS, GM, and HD: writing—original draft preparation; KS, GM, HD, SK, and RV: writing—review and editing; GM and HD: supervision. All authors contributed to the article and approved the submitted version.

## Supporting information


**Figure S1** Genotyping of 230 RILs using InDel marker I‐SP‐383.9.

**Table S1** List of primers used for qRT‐PCR based gene expression analysis.
**Table S2** The InDel position and sequence details of the parents.
**Table S3** InDel primer name and sequence.
**Table S4** List of primers used for cloning of the *LcELF3a* gene.
**Table S5** List of candidate genes identified for days to flowering in the identified QTLs.
**Table S6** Summary of qRT‐PCR results for 11 identified candidate genes from three QTLs on chromosome 3.
**Table S7** Phenotyping and Genotyping data of RIL population for an earliness trait and Single marker analysis.
**Table S7a** Single marker analysis using simple linear regression with two classes (EE/Ee = 1 and ee = 0) for DCM.
**Table S7b** Single marker analysis using simple linear regression with a three‐class scenario (EE = 1, Ee = 2, and ee = 0) for DCM.
**Table S8** Pairwise *LcElf3a* CDS alignment between the late parent (Globe mutant) and early parent (L4775).
**Table S9** Pairwise *LcElf3a* sequence alignment at an amino acid level between the late parent (Globe mutant) and early parent (L4775).

## Data Availability

The data that support the findings of this study are openly available in sequence read archive at https://www.ncbi.nlm.nih.gov/bioproject/PRJNA915231, reference number PRJNA915231.
