# Supplementary material for: Delineation of loci governing an extra‐earliness trait in lentil (Lens culinaris Medik.) using the QTL‐Seq approach
Source: Plant Biotechnol J. 2024 Jun 25;22(10):2932–49. doi: 10.1111/pbi.14415 (PMC11536446; doi:10.1111/pbi.14415)
Supplement: Supplementary file 2 — Table S1 List of primers used for qRT‐PCR based gene expression analysis. Table S2 The InDel position and sequence details of the parents. Table S3 InDel primer name and sequence. Table S4 List of primers used for cloning of the LcELF3a gene. Table S5 List of candidate genes identified for days to flowering in the identified QTLs. Table S6 Summary of qRT‐PCR results for 11 identified candidate genes from three QTLs on chromosome 3. Table S7 Phenotyping and Genotyping data of RIL population for an earliness trait and Single marker analysis. Table S7a Single marker analysis using simple linear regression with two classes (EE/Ee = 1 and ee = 0) for DCM. Table S7b Single marker analysis using simple linear regression with a three‐class scenario (EE = 1, Ee = 2, and ee = 0) for DCM. Table S8 Pairwise LcElf3a CDS alignment between the late parent (Globe mutant) and early parent (L4775). Table S9 Pairwise LcElf3a sequence alignment at an amino acid level between the late parent (Globe mutant) and early parent (L4775). [file PBI-22-2932-s002.docx]

**Table S1. List of primers used for qRT-PCR based gene expression analysis.**

| **Target gene** | **Primer name** | **Sequence** | **Reference** |
| --- | --- | --- | --- |
| *LcActin* | LcActin-Fw | CCAAATCATGTTTGAGGCTTTTAA | Sen Gupta *et al*., 2017 |
|  | LcActin-Rv | GTGAAAGAACGGCCTGAATAGC |  |
| *LcGA20oxG* | GA20-F | CAGGTCCTCACTCTGACCCT |  |
|  | GA20-R | CTAGGGCGAACAGAAAGCCA |  |
| *LcFRI* | FRI-F | CAACCTTCATCGGCACAAGC |  |
|  | FRI-R | ACAAATCAGAGCCTCCACGG |  |
| *LcLFY* | LFY-F | ACTCCTACAACCGCTCCGA |  |
|  | LFY-R | GCTCAGCTATTTTCGCTGCC |  |
| *LcSPL13a* | SPL13a-F | GAAGCGGTTAGACGGACACA |  |
|  | SPL13a-R | TAGCAACTGTGTGCCTTGGT |  |
| *Lcu.2RBY.3g059660* | NF-YC3-F | ACAGCAGCAACAACAACAGC |  |
|  | NF-YC3-R | ACGTCTTCGTCGGCTTTCAT |  |
| *Lcu.2RBY.3g060720* | UbL-cp1F | CCTCTTCGGTTGGTGCATCT |  |
|  | UbL-cp1R | TTCTGAACCACACGCAATGT |  |
| *Lcu.2RBY.3g062540* | F-bGID2F | TTGGTGGGTTTCGTCGTCTT |  |
|  | F-Bgid2r | TTCGACCGAATCACCTGTGG |  |
| *Lcu.2RBY.3g062620* | CCAAT-F | GGCTGATGGACAAGAGCAGT |  |
|  | CCAAT-R | CACCAAGCTGTGGAGGTGAT |  |
| *Lcu.2RBY.3g062760* | MADSb-F | TGCAAACATGGACCGTGACT |  |
|  | MADSb-R | AACATTCGCCCAGTAAGCCA |  |
| *LcELF3a* | ELF3aq1-F | TGGACATGGACAAAGTGACG |  |
|  | ELF3aq1-R | TTTTGGACTGCCTGTCCCG |  |
| *Lcu.2RBY.3g067540* | UCH-F | TGCAGCAGTGGTGGAAAAGA |  |
|  | UCH-R | CTCAGGAATAAGCACCCGCT |  |
| *LcEMF1* | EMF1-F | ATGCAGCTCTCAAACCTTGT |  |
|  | EMF1-R | TTGGTAGGCAGCCATCGAAA |  |
| *LcELF3a* | ELF3aq2-F | CTCCTGATGATGTTGTTGCAGT |  |
|  | ELF3aq2-R | CGATCCGGCAATTAGTTGTT |  |
|  |  |  |  |
|  |  |  |  |

**Table S2. The InDel position and sequence details of the parents.**

| **InDel position** | **Globe mutant sequence** | **length** | **L4775 sequence** | **length** | **InDel size difference** |
| --- | --- | --- | --- | --- | --- |
| 383964202 | ATTTGACTCTATTGAATATAACTGATTTCTCCTACTT | 37 | ATT | 3 | 34 |

**Table S3. InDel primer name and sequence.**

| **Primer name** | **Sequence** |
| --- | --- |
| I-SP-383.9-F | AGCTAAGGCGCAAGTCCAAA |
| I-SP-383.9-R | TGGGTGTGTTCAACCTAGTCG |

**Table S4. List of primers used for cloning of *LcELF3a* gene.**

| **Primer name** | **Sequence (5’-3’)** |
| --- | --- |
| ELF3aF | ACACGACCAATACTTTCCGCT |
| ELF3aR | CCGTTACATGATGGCACACC |
| Elf3-gap-seqF | TCAAGATCGTCGCGTGTTCA |

**Table S5. List of candidate genes identified for days to flowering in the identified QTLs.**

| **QTLs** | **Gene name** | **Start site** | **End site** | **Chromosome** |
| --- | --- | --- | --- | --- |
| *LcqDTF3.1* | *LcGA20oxG (Lcu.2RBY.3g057140)* | 347073115 | 347075413 | Chrom03 |
| *LcqDTF3.1* | *LcFRI (Lcu.2RBY.3g058570)* | 356509008 | 356513131 | Chrom03 |
| *LcqDTF3.1* | *LcLFY (Lcu.2RBY.3g058810)* | 357350393 | 357352318 | Chrom03 |
| *LcqDTF3.1* | *LcSPL13a (Lcu.2RBY.3g059590)* | 361572417 | 361575436 | Chrom03 |
| *LcqDTF3.1* | *Lcu.2RBY.3g059660* (nuclear transcription factor Y subunit C3) | 361854730 | 361857871 | Chrom03 |
| *LcqDTF3.2* | *Lcu.2RBY.3g060720 (Ubiquitin ligase cop1)* | 368144263 | 368144738 | Chrom03 |
| *LcqDTF3.2* | *Lcu.2RBY.3g062540 (F-box GID2-like protein)* | 375998970 | 376000802 | Chrom03 |
| *LcqDTF3.2* | *Lcu.2RBY.3g062620 (CCAAT-binding transcription factor*) | 376524069 | 376524707 | Chrom03 |
| *LcqDTF3.2* | *Lcu.2RBY.3g062640* *(CCAAT-binding transcription factor*) | 376536256 | 376536804 | Chrom03 |
| *LcqDTF3.2* | *Lcu.2RBY.3g062760 (AGL MADS-box transcription factor)* | 376970248 | 376972999 | Chrom03 |
| *LcqDTF3.2* | *LcELF3a (Lcu.2RBY.3g063730)* | 382530247 | 382534690 | Chrom03 |
| *LcqDTF3.3* | *Lcu.2RBY.3g067540* (*Ubiquitin carboxyl-terminal hydrolase*) | 399097289 | 399108239 | Chrom03 |
| *LcqDTF3.3* | *LcEMF1 (Lcu.2RBY.3g068830)* | 404787828 | 404794593 | Chrom03 |

**Table S6. Summary of qRT-PCR results for 11 identified candidate genes from three QTLs on chromosome 3.**

| Genes | P value | Mean of GM-30DAS | Mean of L4775-30DAS | Difference | SE of difference | t ratio | df |
| --- | --- | --- | --- | --- | --- | --- | --- |
| *LcGA20oxG* | 0.003392 | 3.884 | 1.001 | 2.884 | 0.4633 | 6.225 | 4 |
| *LcFRI* | 0.005856 | 1.734 | 1.003 | 0.7311 | 0.1365 | 5.358 | 4 |
| *LcLFY* | 0.000058 | 1.068 | 10.62 | -9.552 | 0.5344 | 17.87 | 4 |
| *LcSPL13a* | 0.030444 | 1.028 | 1.638 | -0.6093 | 0.1856 | 3.282 | 4 |
| *Lcu.2RBY.3g060720* | 0.000043 | 3.515 | 1.005 | 2.51 | 0.1304 | 19.25 | 4 |
| *Lcu.2RBY.3g062540* | 0.000308 | 2.426 | 1.005 | 1.42 | 0.1217 | 11.67 | 4 |
| *Lcu.2RBY.3g062620* | 0.071738 | 1.421 | 1.102 | 0.3185 | 0.1309 | 2.433 | 4 |
| *Lcu.2RBY.3g062760* | 0.001737 | 0.9435 | 1.413 | -0.4697 | 0.06308 | 7.446 | 4 |
| *LcELF3a* | 0.000002 | 25.06 | 1.001 | 24.06 | 0.5648 | 42.6 | 4 |
| *Lcu.2RBY.3g067540* | 0.240363 | 1.314 | 1.008 | 0.3058 | 0.222 | 1.378 | 4 |
| *LcEMF1* | 0.000292 | 1.902 | 1.005 | 0.897 | 0.07577 | 11.84 | 4 |

**Table S7. Phenotyping and Genotyping data of RIL population for earliness trait and Single marker analysis.**

| RIL No. | Genotypic data for InDel-383.9 with 3 class EE=1, Ee=2, ee =0 | Genotypic data for InDel-383.9 with 2 class EE/Ee=1 and ee=0 | Days to first flowering (DTF) | Days to 50% of lines flowers in a row (DFF) | Days to first maturity (DTM) | Days to 50% of lines mature in a row (DFM) | Days to 100% lines matures in a row (DCM) |
| --- | --- | --- | --- | --- | --- | --- | --- |
| 1 | 0 | 0 | 63 | 65 | 103 | 103 | 115 |
| 2 | 0 | 0 | 60 | 60 | 100 | 103 | 115 |
| 3 | 0 | 0 | 56 | 63 | 91 | 96 | 106 |
| 4 | 1 | 1 | 88 | 90 | 119 | 126 | 134 |
| 5 | 2 | 1 | 57 | 63 | 119 | 126 | 134 |
| 6 | 0 | 0 | 60 | 63 | 96 | 102 | 103 |
| 7 | 1 | 1 | 63 | 97 | 134 | 136 | 137 |
| 8 | 1 | 1 | 88 | 93 | 126 | 136 | 140 |
| 9 | 2 | 1 | 60 | 70 | 103 | 136 | 144 |
| 10 | 0 | 0 | 57 | 60 | 91 | 96 | 103 |
| 11 | 0 | 0 | 56 | 60 | 88 | 96 | 103 |
| 12 | 0 | 0 | 60 | 63 | 95 | 96 | 103 |
| 13 | 1 | 1 | 84 | 88 | 119 | 126 | 136 |
| 14 | 2 | 1 | 60 | 91 | 134 | 144 | 144 |
| 15 | 0 | 0 | 60 | 63 | 95 | 103 | 115 |
| 16 | 1 | 1 | 84 | 90 | 116 | 119 | 126 |
| 17 | 1 | 1 | 88 | 93 | 140 | 144 | 144 |
| 18 | 0 | 0 | 56 | 60 | 93 | 96 | 115 |
| 19 | 1 | 1 | 91 | 97 | 140 | 144 | 144 |
| 20 | 0 | 0 | 60 | 63 | 96 | 103 | 109 |
| 21 | 0 | 0 | 60 | 60 | 100 | 103 | 109 |
| 22 | 1 | 1 | 91 | 93 | 126 | 136 | 144 |
| 23 | 1 | 1 | 91 | 93 | 119 | 144 | 144 |
| 24 | 0 | 0 | 60 | 65 | 96 | 103 | 111 |
| 25 | 1 | 1 | 84 | 87 | 119 | 126 | 134 |
| 26 | 2 | 1 | 60 | 63 | 119 | 126 | 134 |
| 27 | 2 | 1 | 60 | 63 | 119 | 126 | 144 |
| 28 | 0 | 0 | 57 | 63 | 91 | 96 | 104 |
| 29 | 1 | 1 | 84 | 88 | 106 | 119 | 140 |
| 30 | 2 | 1 | 57 | 88 | 119 | 136 | 144 |
| 31 | 1 | 1 | 91 | 93 | 126 | 136 | 144 |
| 32 | 1 | 1 | 91 | 93 | 124 | 126 | 134 |
| 33 | 0 | 0 | 55 | 60 | 93 | 96 | 106 |
| 34 | 2 | 1 | 60 | 88 | 106 | 126 | 144 |
| 35 | 0 | 0 | 57 | 60 | 91 | 96 | 103 |
| 36 | 1 | 1 | 88 | 88 | 126 | 134 | 136 |
| 37 | 1 | 1 | 88 | 88 | 134 | 136 | 136 |
| 38 | 2 | 1 | 68 | 73 | 125 | 126 | 136 |
| 39 | 2 | 1 | 56 | 60 | 106 | 119 | 136 |
| 40 | 1 | 1 | 88 | 88 | 119 | 126 | 136 |
| 41 | 2 | 1 | 62 | 73 | 116 | 119 | 126 |
| 42 | 1 | 1 | 88 | 88 | 119 | 126 | 136 |
| 43 | 2 | 1 | 57 | 73 | 112 | 116 | 119 |
| 44 | 2 | 1 | 62 | 70 | 100 | 124 | 126 |
| 45 | 2 | 1 | 57 | 70 | 119 | 125 | 127 |
| 46 | 0 | 0 | 57 | 60 | 92 | 95 | 96 |
| 47 | 1 | 1 | 91 | 93 | 114 | 119 | 136 |
| 48 | 1 | 1 | 73 | 88 | 122 | 125 | 126 |
| 49 | 0 | 0 | 55 | 60 | 91 | 96 | 106 |
| 50 | 1 | 1 | 91 | 96 | 130 | 133 | 134 |
| 51 | 1 | 1 | 91 | 100 | 126 | 136 | 144 |
| 52 | 0 | 0 | 55 | 60 | 96 | 96 | 103 |
| 53 | 1 | 1 | 93 | 97 | 126 | 135 | 137 |
| 54 | 1 | 1 | 91 | 93 | 96 | 128 | 136 |
| 55 | 2 | 1 | 60 | 63 | 100 | 111 | 119 |
| 56 | 1 | 1 | 96 | 100 | 126 | 130 | 137 |
| 57 | 1 | 1 | 91 | 93 | 126 | 130 | 136 |
| 58 | 2 | 1 | 56 | 63 | 96 | 119 | 124 |
| 59 | 2 | 1 | 57 | 63 | 96 | 119 | 136 |
| 60 | 0 | 0 | 56 | 63 | 96 | 103 | 109 |
| 61 | 1 | 1 | 91 | 96 | 126 | 135 | 137 |
| 62 | 0 | 0 | 60 | 65 | 103 | 106 | 112 |
| 63 | 1 | 1 | 96 | 100 | 136 | 144 | 144 |
| 64 | 0 | 0 | 55 | 56 | 88 | 96 | 100 |
| 65 | 2 | 1 | 60 | 70 | 100 | 126 | 136 |
| 66 | 1 | 1 | 91 | 93 | 106 | 126 | 136 |
| 67 | 1 | 1 | 91 | 93 | 119 | 126 | 136 |
| 68 | 0 | 0 | 57 | 60 | 91 | 93 | 100 |
| 69 | 0 | 0 | 60 | 63 | 98 | 100 | 103 |
| 70 | 0 | 0 | 60 | 63 | 98 | 100 | 109 |
| 71 | 0 | 0 | 60 | 63 | 98 | 100 | 103 |
| 72 | 0 | 0 | 57 | 63 | 100 | 103 | 103 |
| 73 | 2 | 1 | 63 | 98 | 106 | 126 | 137 |
| 74 | 0 | 0 | 60 | 63 | 100 | 103 | 109 |
| 75 | 1 | 1 | 98 | 100 | 126 | 136 | 138 |
| 76 | 0 | 0 | 60 | 63 | 93 | 96 | 100 |
| 77 | 0 | 0 | 60 | 63 | 93 | 96 | 100 |
| 78 | 0 | 0 | 60 | 63 | 96 | 96 | 100 |
| 79 | 1 | 1 | 89 | 90 | 112 | 116 | 119 |
| 80 | 2 | 1 | 56 | 57 | 96 | 126 | 136 |
| 81 | 2 | 1 | 57 | 91 | 100 | 114 | 126 |
| 82 | 2 | 1 | 55 | 56 | 100 | 114 | 119 |
| 83 | 0 | 0 | 55 | 56 | 88 | 96 | 100 |
| 84 | 0 | 0 | 60 | 63 | 96 | 98 | 100 |
| 85 | 0 | 0 | 60 | 65 | 93 | 96 | 100 |
| 86 | 0 | 0 | 56 | 60 | 91 | 96 | 115 |
| 87 | 0 | 0 | 60 | 63 | 96 | 100 | 103 |
| 88 | 0 | 0 | 56 | 60 | 88 | 96 | 103 |
| 89 | 0 | 0 | 60 | 63 | 96 | 100 | 106 |
| 90 | 0 | 0 | 60 | 63 | 93 | 96 | 103 |
| 91 | 0 | 0 | 57 | 63 | 88 | 96 | 112 |
| 92 | 0 | 0 | 56 | 63 | 91 | 96 | 113 |
| 93 | 0 | 0 | 55 | 57 | 96 | 116 | 116 |
| 94 | 0 | 0 | 60 | 63 | 88 | 96 | 100 |
| 95 | 0 | 0 | 57 | 63 | 87 | 96 | 112 |
| 96 | 0 | 0 | 57 | 65 | 103 | 111 | 116 |
| 97 | 0 | 0 | 60 | 63 | 100 | 103 | 106 |
| 98 | 0 | 0 | 56 | 60 | 91 | 96 | 100 |
| 99 | 0 | 0 | 57 | 60 | 91 | 96 | 100 |
| 100 | 0 | 0 | 57 | 60 | 98 | 100 | 103 |
| 101 | 1 | 1 | 63 | 68 | 100 | 136 | 144 |
| 102 | 0 | 0 | 57 | 60 | 91 | 96 | 100 |
| 103 | 1 | 1 | 84 | 89 | 114 | 119 | 139 |
| 104 | 0 | 0 | 56 | 60 | 93 | 100 | 109 |
| 105 | 2 | 1 | 57 | 60 | 96 | 141 | 144 |
| 106 | 1 | 1 | 89 | 93 | 114 | 119 | 134 |
| 107 | 2 | 1 | 60 | 63 | 114 | 116 | 119 |
| 108 | 1 | 1 | 84 | 93 | 119 | 129 | 137 |
| 109 | 1 | 1 | 84 | 89 | 119 | 129 | 136 |
| 110 | 1 | 1 | 91 | 91 | 119 | 129 | 136 |
| 111 | 1 | 1 | 89 | 90 | 114 | 119 | 136 |
| 112 | 0 | 0 | 56 | 57 | 91 | 96 | 103 |
| 113 | 1 | 1 | 56 | 60 | 93 | 119 | 136 |
| 114 | 2 | 1 | 57 | 63 | 96 | 119 | 135 |
| 115 | 1 | 1 | 91 | 93 | 129 | 132 | 135 |
| 116 | 2 | 1 | 63 | 84 | 96 | 130 | 133 |
| 117 | 1 | 1 | 91 | 93 | 129 | 133 | 133 |
| 118 | 1 | 1 | 70 | 86 | 119 | 130 | 136 |
| 119 | 0 | 0 | 56 | 63 | 93 | 100 | 103 |
| 120 | 2 | 1 | 55 | 60 | 93 | 119 | 121 |
| 121 | 0 | 0 | 57 | 60 | 100 | 103 | 114 |
| 122 | 0 | 0 | 55 | 56 | 91 | 95 | 96 |
| 123 | 0 | 0 | 56 | 57 | 91 | 95 | 96 |
| 124 | 2 | 1 | 60 | 63 | 114 | 116 | 117 |
| 125 | 0 | 0 | 60 | 63 | 93 | 100 | 100 |
| 126 | 0 | 0 | 58 | 60 | 94 | 96 | 100 |
| 127 | 0 | 0 | 58 | 60 | 93 | 96 | 100 |
| 128 | 0 | 0 | 55 | 57 | 94 | 95 | 96 |
| 129 | 0 | 0 | 56 | 63 | 93 | 96 | 100 |
| 130 | 0 | 0 | 60 | 63 | 93 | 96 | 100 |
| 131 | 0 | 0 | 58 | 63 | 96 | 100 | 106 |
| 132 | 0 | 0 | 58 | 63 | 98 | 100 | 115 |
| 133 | 0 | 0 | 56 | 63 | 96 | 100 | 106 |
| 134 | 0 | 0 | 60 | 63 | 96 | 100 | 109 |
| 135 | 0 | 0 | 56 | 60 | 91 | 100 | 106 |
| 136 | 0 | 0 | 63 | 63 | 100 | 103 | 106 |
| 137 | 0 | 0 | 63 | 65 | 100 | 103 | 106 |
| 138 | 0 | 0 | 55 | 56 | 91 | 96 | 100 |
| 139 | 0 | 0 | 60 | 63 | 97 | 103 | 144 |
| 140 | 0 | 0 | 57 | 63 | 100 | 103 | 106 |
| 141 | 0 | 0 | 57 | 63 | 91 | 96 | 106 |
| 142 | 0 | 0 | 63 | 65 | 100 | 103 | 106 |
| 143 | 0 | 0 | 60 | 63 | 96 | 100 | 103 |
| 144 | 0 | 0 | 60 | 63 | 96 | 100 | 103 |
| 145 | 0 | 0 | 60 | 63 | 93 | 100 | 103 |
| 146 | 0 | 0 | 57 | 63 | 93 | 96 | 103 |
| 147 | 0 | 0 | 60 | 63 | 94 | 96 | 106 |
| 148 | 1 | 1 | 63 | 68 | 96 | 136 | 137 |
| 149 | 0 | 0 | 60 | 63 | 93 | 96 | 103 |
| 150 | 2 | 1 | 56 | 60 | 96 | 119 | 138 |
| 151 | 2 | 1 | 60 | 73 | 96 | 129 | 139 |
| 152 | 0 | 0 | 60 | 65 | 98 | 100 | 103 |
| 153 | 2 | 1 | 93 | 96 | 129 | 144 | 144 |
| 154 | 2 | 1 | 56 | 60 | 96 | 119 | 136 |
| 155 | 2 | 1 | 57 | 68 | 136 | 144 | 144 |
| 156 | 2 | 1 | 56 | 68 | 106 | 136 | 144 |
| 157 | 2 | 1 | 60 | 91 | 119 | 134 | 136 |
| 158 | 2 | 1 | 60 | 68 | 112 | 119 | 129 |
| 159 | 1 | 1 | 89 | 89 | 111 | 119 | 129 |
| 160 | 0 | 0 | 60 | 68 | 96 | 100 | 103 |
| 161 | 1 | 1 | 57 | 68 | 110 | 116 | 119 |
| 162 | 0 | 0 | 60 | 65 | 100 | 102 | 103 |
| 163 | 0 | 0 | 65 | 65 | 96 | 100 | 103 |
| 164 | 2 | 1 | 60 | 63 | 100 | 119 | 136 |
| 165 | 2 | 1 | 57 | 63 | 104 | 119 | 129 |
| 166 | 1 | 1 | 96 | 98 | 129 | 136 | 144 |
| 167 | 1 | 1 | 63 | 84 | 109 | 114 | 118 |
| 168 | 2 | 1 | 60 | 84 | 112 | 116 | 119 |
| 169 | 1 | 1 | 84 | 87 | 119 | 130 | 136 |
| 170 | 0 | 0 | 63 | 63 | 96 | 100 | 103 |
| 171 | 1 | 1 | 93 | 97 | 129 | 133 | 136 |
| 172 | 1 | 1 | 60 | 63 | 106 | 130 | 136 |
| 173 | 0 | 0 | 60 | 63 | 103 | 105 | 106 |
| 174 | 1 | 1 | 94 | 95 | 129 | 133 | 136 |
| 175 | 1 | 1 | 100 | 103 | 129 | 133 | 136 |
| 176 | 0 | 0 | 63 | 65 | 98 | 100 | 106 |
| 177 | 0 | 0 | 60 | 63 | 98 | 100 | 103 |
| 178 | 2 | 1 | 57 | 63 | 98 | 109 | 117 |
| 179 | 2 | 1 | 55 | 68 | 96 | 109 | 117 |
| 180 | 0 | 0 | 58 | 60 | 93 | 96 | 103 |
| 181 | 2 | 1 | 57 | 63 | 112 | 114 | 119 |
| 182 | 1 | 1 | 84 | 87 | 119 | 129 | 135 |
| 183 | 0 | 0 | 63 | 65 | 103 | 106 | 119 |
| 184 | 2 | 1 | 56 | 56 | 100 | 114 | 129 |
| 185 | 0 | 0 | 60 | 63 | 96 | 100 | 103 |
| 186 | 0 | 0 | 60 | 63 | 96 | 100 | 104 |
| 187 | 1 | 1 | 84 | 87 | 119 | 129 | 136 |
| 188 | 1 | 1 | 89 | 91 | 129 | 135 | 137 |
| 189 | 0 | 0 | 60 | 63 | 96 | 103 | 103 |
| 191 | 1 | 1 | 57 | 60 | 119 | 136 | 144 |
| 192 | 2 | 1 | 60 | 63 | 113 | 116 | 136 |
| 193 | 1 | 1 | 90 | 91 | 129 | 136 | 137 |
| 194 | 1 | 1 | 89 | 91 | 129 | 135 | 137 |
| 195 | 1 | 1 | 90 | 93 | 129 | 135 | 136 |
| 196 | 1 | 1 | 84 | 84 | 112 | 119 | 136 |
| 197 | 0 | 0 | 60 | 63 | 91 | 96 | 100 |
| 198 | 0 | 0 | 60 | 63 | 88 | 96 | 115 |
| 199 | 2 | 1 | 56 | 60 | 114 | 119 | 136 |
| 200 | 2 | 1 | 56 | 63 | 112 | 119 | 129 |
| 201 | 1 | 1 | 57 | 91 | 129 | 133 | 136 |
| 202 | 1 | 1 | 89 | 90 | 129 | 135 | 135 |
| 203 | 1 | 1 | 57 | 90 | 119 | 129 | 136 |
| 204 | 0 | 0 | 60 | 63 | 91 | 100 | 103 |
| 205 | 2 | 1 | 60 | 63 | 91 | 136 | 144 |
| 206 | 0 | 0 | 56 | 57 | 88 | 93 | 103 |
| 207 | 0 | 0 | 56 | 60 | 88 | 93 | 103 |
| 208 | 1 | 1 | 57 | 63 | 91 | 136 | 144 |
| 209 | 1 | 1 | 86 | 89 | 114 | 116 | 119 |
| 210 | 2 | 1 | 63 | 68 | 100 | 120 | 129 |
| 211 | 1 | 1 | 91 | 93 | 129 | 135 | 137 |
| 212 | 2 | 1 | 60 | 63 | 109 | 114 | 136 |
| 213 | 0 | 0 | 60 | 63 | 91 | 100 | 103 |
| 214 | 1 | 1 | 84 | 93 | 133 | 136 | 138 |
| 215 | 0 | 0 | 60 | 63 | 93 | 100 | 103 |
| 216 | 0 | 0 | 57 | 63 | 91 | 100 | 103 |
| 217 | 0 | 0 | 56 | 60 | 88 | 93 | 96 |
| 218 | 0 | 0 | 60 | 65 | 88 | 93 | 96 |
| 219 | 2 | 1 | 60 | 65 | 91 | 130 | 136 |
| 220 | 0 | 0 | 57 | 63 | 91 | 100 | 109 |
| 222 | 0 | 0 | 57 | 60 | 91 | 96 | 103 |
| 223 | 0 | 0 | 57 | 63 | 93 | 96 | 103 |
| 224 | 0 | 0 | 63 | 63 | 96 | 100 | 103 |
| 225 | 0 | 0 | 57 | 63 | 98 | 100 | 103 |
| 226 | 0 | 0 | 60 | 63 | 96 | 100 | 103 |
| 227 | 0 | 0 | 60 | 63 | 96 | 100 | 103 |
| 228 | 0 | 0 | 60 | 63 | 96 | 100 | 103 |
| 229 | 0 | 0 | 60 | 63 | 96 | 100 | 103 |
| 230 | 0 | 0 | 57 | 63 | 96 | 101 | 103 |
| 231 | 0 | 0 | 59 | 63 | 100 | 100 | 103 |
| 232 | 0 | 0 | 56 | 57 | 88 | 96 | 103 |

**Table S7a. Single marker analysis using simple linear regression with two classes (EE/Ee=1 and ee=0) for DCM**

| Regression Statistics | |  |  |  |  |  |  |  |  |
| --- | --- | --- | --- | --- | --- | --- | --- | --- | --- |
| Multiple R | 0.9075 |  |  |  |  |  |  |  |  |
| R Square | 0.823557 |  |  |  |  |  |  |  |  |
| Adjusted R Square | 0.822783 |  |  |  |  |  |  |  |  |
| Standard Error | 6.855099 |  |  |  |  |  |  |  |  |
| Observations | 230 |  |  |  |  |  |  |  |  |
|  |  |  |  |  |  |  |  |  |  |
| ANOVA |  |  |  |  |  |  |  |  |  |
|  | df | SS | MS | F | Significance F |  |  |  |  |
| Regression | 1 | 50009.43 | 50009.43 | 1064.203 | 7.54E-88 |  |  |  |  |
| Residual | 228 | 10714.26 | 46.99238 |  |  |  |  |  |  |
| Total | 229 | 60723.69 |  |  |  |  |  |  |  |
|  |  |  |  |  |  |  |  |  |  |
|  | Coefficients | Standard Error | t Stat | P-value | Lower 95% | Upper 95% | Lower 95.0% | Upper 95.0% | Upper 95.0% |
| Intercept | 104.9474 | 0.642039 | 163.4595 | 2.8E-238 | 103.6823 | 106.2125 | 103.6823 | 106.2125 | 110.177 |
| X Variable 1 | 29.49229 | 0.904058 | 32.62212 | 7.54E-88 | 27.71091 | 31.27366 | 27.71091 | 31.27366 | 17.62596 |

**Table S7b. Single marker analysis using simple linear regression with three-class scenario (EE=1, Ee=2, and ee=0) for DCM**

| Regression Statistics | |  |  |  |  |  |  |  |
| --- | --- | --- | --- | --- | --- | --- | --- | --- |
| Multiple R | 0.778266 |  |  |  |  |  |  |  |
| R Square | 0.605698 |  |  |  |  |  |  |  |
| Adjusted R Square | 0.603969 |  |  |  |  |  |  |  |
| Standard Error | 10.24769 |  |  |  |  |  |  |  |
| Observations | 230 |  |  |  |  |  |  |  |
|  |  |  |  |  |  |  |  |  |
| ANOVA |  |  |  |  |  |  |  |  |
|  | df | SS | MS | F | Significance F |  |  |  |
| Regression | 1 | 36780.23 | 36780.23 | 350.2373 | 5.68E-48 |  |  |  |
| Residual | 228 | 23943.46 | 105.0152 |  |  |  |  |  |
| Total | 229 | 60723.69 |  |  |  |  |  |  |
|  |  |  |  |  |  |  |  |  |
|  | Coefficients | Standard Error | t Stat | P-value | Lower 95% | Upper 95% | Lower 95.0% | Upper 95.0% |
| Intercept | 108.3815 | 0.911192 | 118.9447 | 3.6E-207 | 106.5861 | 110.177 | 106.5861 | 110.177 |
| X Variable 1 | 15.94694 | 0.852111 | 18.71463 | 5.68E-48 | 14.26792 | 17.62596 | 14.26792 | 17.62596 |

**Table S8. Pairwise *LcElf3a* CDS alignment between the late parent (Globe mutant) and early parent (L4775)**

Late    ACACGACCAATACTTTCCGCTTCTTTCTTCACAAGAACAAAACAAGAATCCCTAATTTTTCT------CTTCACT
Early   ACACGACCAATACTTTCCGCTTCTTTCTTCACAAGAACAAAACAAGAATCCCTAATTTTTCTCTTTCTCTTCACT

cons    **************************************************************      *******

Late    TTTCAATCTTTCTCTCACAATTTGATTTTCTTTCTCTAGTTGTAATCTAAATCAACTGTGAATTT-TAGGTTTTT
Early   TTTCAATCTTTCTTTCACAATTTGATTTTCTTTCTCCAGTTGTAATCTAGATCAACTGTGAATTTTTAGGTTTTT

cons    ************* ********************** ************ *************** *********

Late    CTGGTAAATAGTTGGGAGGAGTTTTTAGATTTGGTGAATTGGATGTGTCTGTGCCAGCTATAATCACTTTTTTTT
Early   CTGGTAAATAGTTGGGAGGAGTTTTTAGATTTGGTGAATTGGATGTGTCTGTGCCAGCCATAATCACTTTTTTTT

cons    ********************************************************** ****************

Late    TAGCTTCAGAATTATAAGTGATTGTGTTGTGGAAGTGTAATTGAGTTGTTGTATTAGAGGATTATGCTTTTGTTG
Early   TAGCTTCAGAATTATAAGTGATTGTGTTGTGGAAGTGTAATTGAGTTGTTGTATTAGAGGATTGTGCTTTTGTTG

cons    *************************************************************** ***********

Late    TGTGTTTAGAGTTTAAGATAGAAAAGGGGTAGGGTTTTGTTTGTTGTGTTAGGGAAGGTTA-GGATGAAAAGAGG
Early   TGTGTTTAGAGTTTAAGATAGAAAAGGGGTAGGGTTTTGTTTGTTGTGTTAGGGAAGGTTAAGGATGAAAAGAGG

cons    ************************************************************* *************

Late    GAGTGATGATGAGAAAATGATGGGGCCATTATTTCCTAGATTACATGTTGGTGATACAGAGAAAGGAGGGCCTAG
Early   GAGTGATGATGAGAAAATGATGGGGCCATTATTTCCTAGATTACATGTTGGTGATACAGAGAAAGGAGGGCCTAG

cons    ***************************************************************************

Late    AGCACCACCTAGGAATAAAATGGCTTTATATGAGCAGTTTAGTATTCCTTCTCAAAGGTTTAACTTGCCACTACA
Early   AGCACCACCTAGGAATAAAATGGCTTTATATGAGCAGTTTAGTATTCCTTCTCAAAGGTTTAACTTGCCACTACA

cons    ***************************************************************************

Late    CCCTAATAATTCGACCAATTCGGTTCCCCCGGCTTCCTCGAGCCAGGGAACTGTCCATGAGCGAAACTATATTTT
Early   CCCTAATAATTCGACCAATTCGGTTCCCCCGGCTTCCTCGAGCCAGGGGACTGTCCATGAGCGAAACTATATTTT

cons    ************************************************ **************************

Late    TCCGGGTCATTTGACGCCTGAAACACTTATTCGTCAGGCTGGAAAACATCTCTCTCGCCAATCAAAAGGGGCAAA
Early   TCCTGGTCATTTGACGCCTGAAACACTTATTCGTCAGGCTGGAAAACATCTCTCTCGCCAATCAAAAGGGGCAAA

cons    *** ***********************************************************************

Late    TTTGAATGGTTCTATAGCACAAATTGAACATAGAAAGAAGGTTGACGAAGATGACTTTAGGGTTCCCGTATACGT
Early   TTTGAATGGTTCTATAGCACAAATTGAACATAGAAAGAAGGTTGATGAAGATGACTTTAGGGTTCCTGTATACGT

cons    ********************************************* ******************** ********

Late    TCGTTCAAATATTGGTCAATCTAATGAGAAAAGGCCTGAGAGTTTTGATGGGAAAAGACCCCCTTCTACAGGCTC
Early   TCGTTCAAATATTGGTCAATCTAATGAGAAAAGGCCTGAGAGTTTTGATGGGAAAAGACCCCCTTCTACAGGCTC

cons    ***************************************************************************

Late    TAGGTATTTTGGTTTTTTGAAGCCAGGTAAGATTGATCGGGAGAGGGAGCTGATACAGAATGGCTCCACAGTTGT
Early   TAGGTATTTTGGTTTTTTGAAGCCAGGTAAGATTGATCGGGAGAGGGAGCTGATACAGAATGGCTCCACAGTTGT

cons    ***************************************************************************

Late    TAATGCAGGGACAGACGTGAGAAATGAGATTGATGGTCCTCCGCAAGTGAGTCCAAATAAGGAGCATCCGTTTAC
Early   TAATGCAGGGACAGACGTGAGAAATGAGATTGATGGTCCTCCGCAAGTGAGTCCAAATAAGGAGCACCCGGTTAC

cons    ****************************************************************** *** ****

Late    GTCTGCCAGGAACGAATCGACTGGAGAACGTGTTGATGCCTTAGTAAGACAAGTCAAAGTGACTCCAAATCAAGA
Early   GTCTGCCAGGAACGAATCGACTGGAGAACGTGTTGATGCCTTAGTAAGACAAGTCAAAGTGACTCCAAATCAAGA

cons    ***************************************************************************

Late    GGTTCAAGATCGTCGCGTGTTCAAACATAGCAGTTTACGTCAAGGTGATGCACGCTTACGACATGATTGCCGAGC
Early   GGTTCAAGATCGTCGCGTGTTCAAACATAGCAGTTTACGTCAAGGTGATGCACGCTTACGACAAGATTGCCGAGC

cons    *************************************************************** ***********

Late    TGAGTCTCAATCCAATGGACATGGACAAAGTGACGGTCTCCTTGAATCTACAAGGGAGGTAGACATGAGTAATGG
Early   TGAGTCTCAATCCAATGGACATGGACAAAGTGACGGTCTCCTTGAATCTACAAGGGAGGTAGACACGAGTAATGG

cons    ***************************************************************** *********

Late    CCCTATAGTAAACCAAATCAGTCCAACCCAGGCTATCAATGGCACTGAATATCATGATACCGGGACAGGTAGTCC
Early   CCCTATAGTAAACCAAATCAGTCCAACCCAGGCTATCAATGACACTGAATATCATGATACCGGGACAGGCAGTCC

cons    ***************************************** *************************** *****

Late    AAAACAGTTAGGAAATTTAAACAAAAATGACAACATTTCCAAGATCTCCAGGGTAGAAAATTTGTCAACTGTGAA
Early   AAAACAGTTAGGAAATTTAAACAAAAAT---AACATTTCCAAGATCTCCAGGGTAGAAAATTTGTCAACTGTGAA

cons    ****************************   ********************************************

Late    AATTTCTCCTGATGATGTTGTTGCAGTAATAGGCCAAAAACATTTCTGGAAAGCCAGAAAAGCAATTGCCAATCA
Early   AATTTCTCCTGATGATGTTGTTGCAGTAATAGGCCAAAAACATTTCTGGAAAGCCAGAAAAGCAATTGCCAA---

cons    ************************************************************************   

Late    ACAGAGAGTGTTTGCAGTACAAGTGTTTGAGTTGCATAGACTGATAAAGGTCCAACAACTGATTGCCGGATCGCC
Early   -------------------------------------------------GTCCAACAACTGATTGCCGGATCGCC

cons                                                     **************************

Late    AGATCTATTGTTTGACGATGGTGCTTTTTTAGGAAAGTCTCTTCCAGATGGATCTACTCCTAAAAAACTCCCATT
Early   AGATCTATTGTTTGACGATGGTGCTTTTTTAGGAAAGTCTCTTCCAGATGGATCTACTCCTAAAAAACTCCCATT

cons    ***************************************************************************

Late    GGAATATGTTGTAAAAACTCGGCTACAAAATCTTAAGCGCAAAGTTGATTCTGAAAAGATAAATCAAAACATGGA
Early   GGAATATGTTGTAAAAACTCGGCTACAAAATCTTAAGCGCAAAGTTGATTCTGAAAAGATAAATCAAAACATGGA

cons    ***************************************************************************

Late    ATGTTCTGCAGAGAATGCTGTTGGTAAAACATCTATTTCGTCTGTGAAAAATACGAGCCACCTTTCTAGTTCCAT
Early   ATGTTCTGCAGAGAATGCTGTTGGTAAAACATCTATTTCGTCTGTGAAAAATACGAGCCACCTTTCTAGTTCCAT

cons    ***************************************************************************

Late    GCCTTTTGCCGGAAATCCACACCAAGGAAATATGGCTGCTGATAATGGGATGGGTCCCTGGTGTTTCAATCAGTC
Early   GCCTTTTGCCGGAAATCCACACCAAGGAAATATGGCTGCTGATAATGGGATGGGTCCCTGGTGTTTCAATCAGTC

cons    ***************************************************************************

Late    ACCTGGGCATCAGTGGTTAATTCCCGTGATGTCTCCTTCTGAAGGGCTCGTCTACAAGCCATATCCCGGGCCTGG
Early   ACCTGGGCATCAGTGGTTAATTCCCGTGATGTCTCCTTCTGAAGGGCTCGTCTACAAGCCATATCCCGGGCCTGG

cons    ***************************************************************************

Late    ATTTACCGGAACAAATTTTGGAGGATGTGGGCCCTATGGGGCTTCTCCTTCGGGTGGCACTTTTATGAATCCTTC
Early   ATTTACCGGAACAAATTTTGGAGGATGTGGGCCCTATGGGGCTTCTCCTTCGGGTGGCACTTTTATGAATCCTTC

cons    ***************************************************************************

Late    CTATGGAATCCCACCTCCACCAGAGATTCCTCCAGGCAGCCATGCTTACTTCCCTCCGTATGGCGGCATGCCAGT
Early   CTATGGAATCCCACCTCCACCAGAGATTCCTCCAGGCAGCCATGCTTACTTCCCTCCGTATGGCGGCATGCCAGT

cons    ***************************************************************************

Late    TATGAAAGCTGCAGCTTCAGAGTCAGCTGTTGAACATGTGAACCAATTCTCCGCACATGGGCAAAATCACCATTT
Early   TATGAAAGCTGCAGCTTCAGAGTCAGCTGTTGAACATGTGAACCAATTCTCCGCACATGGGCAAAATCACCGTTT

cons    *********************************************************************** ***

Late    ATCTGAAGACGAAGATAATTGTAACAAACATAATCAAAGCTCATGCAATTTACCAGCTCAGAGAAATGAAGATAC
Early   ATCTGAAGACGAAGATAATTGTAACAAACATAATCAAAGCTCATGCAATTTACCAGCTCAGAGAAATGAAGATAC

cons    ***************************************************************************

Late    ATCACATGTCATGTATCATCAGAGATCGAAGGAGTTTGATTTGCAGATGAGTACTGCCAGTAGTCCTAGTGAAAT
Early   ATCACATTTCATGTATCATCAGAGATTGAAGGAGTTTGATTTGCAGATGAGTACCGCCAGTAGTCCTAGTGAAAT

cons    ******* ****************** *************************** ********************

Late    GGCACAAGAAATGAGCACGGGGCAAGTTGCTGAAGGAAGAGATGTACTACCACTTTTCCCTATGGTTTCAGCAGA
Early   GGCACAAGAAATGAGCACGGGGCAAGTTGCTGAAGGAAGAGATGTACTACCACTTTTCCCTATGGTTTCAGCAGA

cons    ***************************************************************************

Late    ACCAGAGAGTGTACCTCATTCTCTCGAAACAGGACAGCAAACTCGAGTTATCAAAGTGGTACCTCATAACCGAAG
Early   ACCAGAGAGTGTACCTCATTCTCTCGAAACAGGACAGCAAACTCGAGTTATCAAAGTGGTACCTCATAACCGAAG

cons    ***************************************************************************

Late    ATCTGCAACTGAATCAGCAGCTAGAATTTTCCAATCAATTCAAGAAGAGAGAAAACAATACGACGCACCCTAGTG
Early   ATCTGCAACTGAATCAGCAGCTAGAATTTTCCAATCAATTCAAGAAGAGAGAAAACAATACGACGCACCCTAGTG

cons    ***************************************************************************

Late    ATATTTATTGGCATGGAGGATCCATTCAAGTGCATGTCGCCTAGTTCCTGCACGTTCAGCAGGTGTGCCATCATG
Early   ATATTTATTGGCATGGAGGATCCATTCAAGTGCATGTCGCCTAGTTCCTGCACGTTCAGCAGGTGTGCCATCATG

cons    ***************************************************************************

Late    TAACGG
Early   TAACGG

cons    ******

**Table S9. Pairwise *LcElf3a* sequence alignment at amino acid level between the late parent (Globe mutant) and early parent (L4775)**

Late    MKRGSDDEKMMGPLFPRLHVGDTEKGGPRAPPRNKMALYEQFSIPSQRFNLPLHPNNSTNSVPPASSSQGTVHER
Early   MKRGSDDEKMMGPLFPRLHVGDTEKGGPRAPPRNKMALYEQFSIPSQRFNLPLHPNNSTNSVPPASSSQGTVHER

cons    ***************************************************************************

Late    NYIFPGHLTPETLIRQAGKHLSRQSKGANLNGSIAQIEHRKKVDEDDFRVPVYVRSNIGQSNEKRPESFDGKRPP
Early   NYIFPGHLTPETLIRQAGKHLSRQSKGANLNGSIAQIEHRKKVDEDDFRVPVYVRSNIGQSNEKRPESFDGKRPP

cons    ***************************************************************************

Late    STGSRYFGFLKPGKIDRERELIQNGSTVVNAGTDVRNEIDGPPQVSPNKEHPFTSARNESTGERVDALVRQVKVT
Early   STGSRYFGFLKPGKIDRERELIQNGSTVVNAGTDVRNEIDGPPQVSPNKEHPVTSARNESTGERVDALVRQVKVT

cons    ****************************************************.**********************

Late    PNQEVQDRRVFKHSSLRQGDARLRHDCRAESQSNGHGQSDGLLESTREVDMSNGPIVNQISPTQAINGTEYHDTG
Early   PNQEVQDRRVFKHSSLRQGDARLRQDCRAESQSNGHGQSDGLLESTREVDTSNGPIVNQISPTQAINDTEYHDTG

cons    ************************:************************* ****************.*******

Late    TGSPKQLGNLNKNDNISKISRVENLSTVKISPDDVVAVIGQKHFWKARKAIANQQRVFAVQVFELHRLIKVQQLI
Early   TGSPKQLGNLNKN-NISKISRVENLSTVKISPDDVVAVIGQKHFWKARKAIAKSNN-------------------

cons    ************* **************************************:.:.                   

Late    AGSPDLLFDDGAFLGKSLPDGSTPKKLPLEYVVKTRLQNLKRKVDSEKINQNMECSAENAVGKTSISSVKNTSHL
Early   ---------------------------------------------------------------------------

cons                                                                               

Late    SSSMPFAGNPHQGNMAADNGMGPWCFNQSPGHQWLIPVMSPSEGLVYKPYPGPGFTGTNFGGCGPYGASPSGGTF
Early   ---------------------------------------------------------------------------

cons                                                                               

Late    MNPSYGIPPPPEIPPGSHAYFPPYGGMPVMKAAASESAVEHVNQFSAHGQNHHLSEDEDNCNKHNQSSCNLPAQR
Early   ---------------------------------------------------------------------------

cons                                                                               

Late    NEDTSHVMYHQRSKEFDLQMSTASSPSEMAQEMSTGQVAEGRDVLPLFPMVSAEPESVPHSLETGQQTRVIKVVP
Early   ---------------------------------------------------------------------------

cons                                                                               

Late    HNRRSATESAARIFQSIQEERKQYDAP
Early   ---------------------------

cons

**
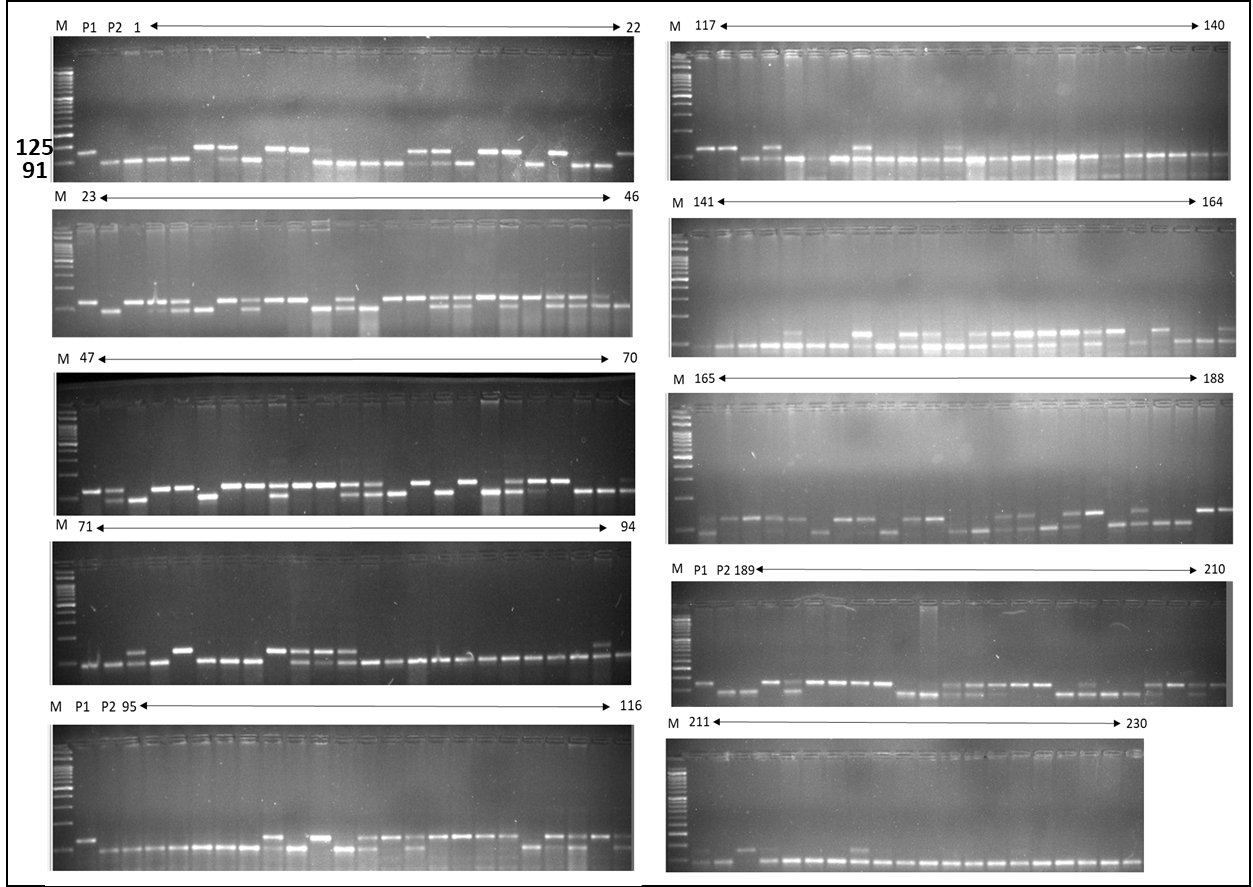
Figure S1. Genotyping of 230 RILs using InDel marker I-SP-383.9.**
