# Supplementary material for: Delineation of loci governing an extra‐earliness trait in lentil (Lens culinaris Medik.) using the QTL‐Seq approach
Source: Plant Biotechnol J. 2024 Jun 25;22(10):2932–49. doi: 10.1111/pbi.14415 (PMC11536446; doi:10.1111/pbi.14415)
Supplement: Supplementary file 2 [file PBI-22-2932-s001.docx]

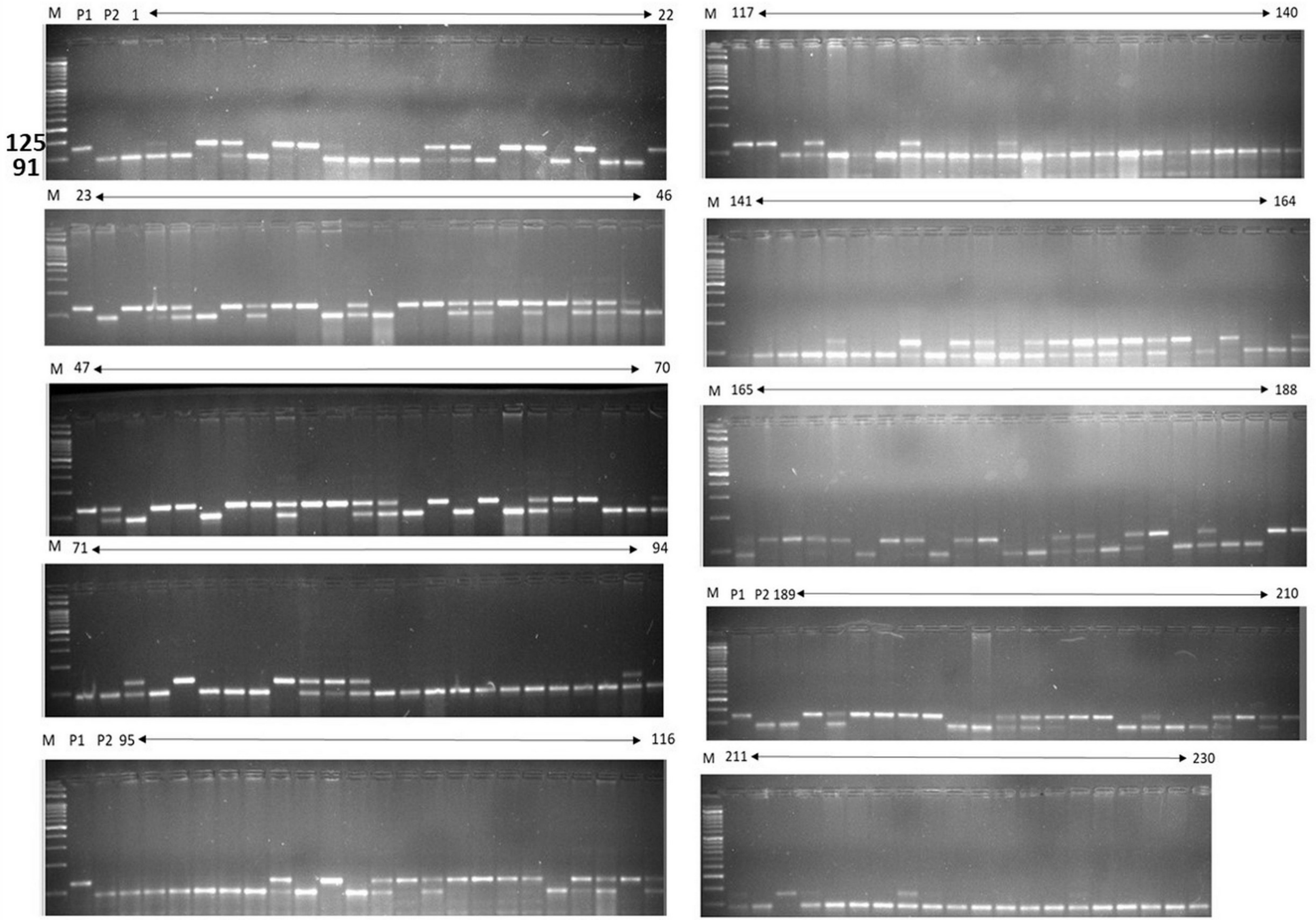

M P1 P2 1 22
125
91
M 23 46
M 47 70
M 71 94
M P1 P2 95 116
M 117 140
M 141 164
M 165 188
M P1 P2 189 210
M 211 230
